# Experimental insights in taxon-specific functional responses to droughts in glacier-fed stream biofilms

**DOI:** 10.1186/s40168-026-02336-6

**Published:** 2026-02-11

**Authors:** David Touchette, Grégoire Michoud, Martin Boutroux, Martina Gonzalez Mateu, Florian Baier, Ianina Altshuler, Hannes Peter, Tom J. Battin

**Affiliations:** 1 River Ecosystems Laboratory, Alpine and Polar Environmental Research Center, School of Architecture, Civil and Environmental Engineering, École Polytechnique Fédérale de Lausanne, Sion, Switzerland; 2 Microbiome Adaptation to the Changing Environment Laboratory, Alpine and Polar Environmental Research Center, School of Architecture, Civil and Environmental Engineering, École Polytechnique Fédérale de Lausanne, Sion, Switzerland

**Keywords:** Biofilm, Glacier-fed stream, Climate change, Drought, Multi-omics

## Abstract

**Background:**

Glacier-fed streams are predicted to face increasingly frequent and intense droughts. However, the impacts of drought events on benthic biofilm, including bacteria, eukaryotes, and viruses, the dominating life form in glacier-fed streams, remain poorly understood.

**Results:**

Using streamside flume mesocosms in the Swiss Alps, we grew glacier-fed stream biofilms over 103 days and exposed them to three droughts. Using a multi-omics approach (metagenomics, metatranscriptomics, and metaproteomics), we assessed the effects of a series of droughts on the taxonomy and metabolic activity of bacterial, eukaryotic, and viral metagenome-assembled genomes (MAGs). We found that the first drought (6 h) caused only minor changes, including mild upregulation of heterotrophic metabolism and signs of stress in diatoms. In contrast, the second drought (24 h) significantly altered both the composition and functionality of the microbiome, shifting phototrophic dominance from diatoms to Cyanobacteriota, while maintaining overall phototropic biomass and further upregulating the heterotrophic metabolism. Interestingly, a third 24 h drought had no detectable transcriptomic effect between pre- and post-drought conditions, suggesting a certain level of adaptive responses to droughts, but with the low diatom abundance being maintained.

**Conclusions:**

These findings indicate that glacier-fed biofilm microorganisms initially resisted short-term drought, but a second longer drought caused important shifts in their community structure, activity, and function. Climate-induced increases in drought frequency or duration may therefore have a lasting impact on microbial ecosystem functioning in glacier-fed streams.

Video Abstract

**Supplementary Information:**

The online version contains supplementary material available at 10.1186/s40168-026-02336-6.

## Background

Glacier-fed streams (GFSs) transport water, sediments, and nutrients to downstream aquatic ecosystems [1], supply freshwater for a large proportion of the population [2], and host diverse bacterial, eukaryotic, and viral communities [3–6]. The glacier-fed stream environment is highly dynamic, varying both diurnally and annually, with marked fluctuations in temperature, light, and nutrient availability [7, 8]. These streams are generally nutrient-poor, cold, and subject to prolonged periods of darkness or intense UV radiation. Despite these harsh conditions, the phenology of glacier-fed streams creates unique seasonal conditions that allow complex biofilm to develop [7, 8].

Glacier-fed streams are also prone to diurnal and seasonal variation in discharge [9, 10], often resulting in lateral contraction of the river network [11, 12]. This exposes stream margins to recurrent drying and rewetting cycles or even complete channel disconnection. Although such flow intermittency is natural in alpine fluvial networks under glacier influence, climate change-induced reduction in snowpack and acceleration of melt [13, 14] are predicted to amplify drought frequency and duration in glacier-fed streams [15–17]. Because benthic biofilms play key roles in ecosystem metabolism and nutrient cycling, and dominate microbial life in these ecosystems [18], understanding their response to drought is critical for anticipating how glacier-fed streams will function under ongoing climate change.

Drought events and hydric stress are increasingly recognized as major ecosystem stressors. Exposure to these environmental stresses imposes physiological challenges on microbial freshwater communities, potentially disrupting ecosystem functions [19]. To maintain functional and compositional stability after a disturbance, communities must exhibit one or more key ecological responses [20, 21]: they may be resistant (insensitive to stress), resilient (able to recover after being disturbed), or functionally redundant (undergo compositional changes but maintain functionality). These ecological responses can provide insights into how drought impacts microorganisms in ecosystems vulnerable to hydrological changes, such as glacier-fed streams.

Recent studies on stream biofilms have shown drought-induced shifts in taxonomic composition [22, 23], a reduction in net primary production and enhancement of heterotrophy [24, 25], but little change in functional potential [23]. Distinct resistance and resilience patterns among microbial taxa were also reported, while bacteria tend to be drought resistant, eukaryotes have stronger recovery after rewetting [22, 25, 26]. Although glacier-fed stream biofilms remain less studied than other freshwater biofilms, recent evidence suggests that their microbiomes exhibit functional redundancy [5], and that their microbial composition is altered by droughts [27]. However, it is unknown how individual clades within glacier-fed stream biofilms actively respond to drought and how this contributes to the overall ecosystem functions of these freshwater ecosystems. Furthermore, identifying the activity patterns of microbial groups within biofilms may provide insights into the resistance, resilience, or drought susceptibility of different microbial taxa within glacier-fed stream biofilms.

In this study, we combined streamside flume mesocosms directly connected to a glacier-fed stream in the Swiss Alps and applied a multi-omics approach to determine the influence of drought on glacier-fed stream biofilm multi-domain composition, metabolic activity, and resistance/resilience patterns. Biofilms grown in these flumes were subjected to three droughts of varying duration. To reveal the diversity within these glacier-fed stream biofilms, we recovered prokaryotic, eukaryotic, and viral DNA metagenome-assembled genomes (MAGs) from metagenomes across the experimental period, and viral RNA MAGs from transcriptomes. We then analysed the impact of drought on compositional and successional patterns in the MAG community. Finally, we used metatranscriptomics combined with protein extraction and identification to capture the functional activity of bacterial, eukaryotic, and viral MAGs, and to assess taxon-specific responses before and after each drought. We hypothesize that drought drives contrasting transcriptional responses in glacier-fed stream biofilm across domains. More precisely, post-drought conditions will stimulate transcriptional activity of resistant bacteria, whereas drought-susceptible eukaryotes will show reduced activity alongside an increase in stress response pathways. We expect drought to decrease the eukaryotic phototrophic activity, accompanied by an increased expression of photosynthesis and carbon-fixation pathways in phototrophic bacteria, since freshwater algae are more sensitive to drying than cyanobacteria [28], thus indicating functional redundancy within the biofilm microbiome. We also hypothesized that viruses contribute to glacier-fed stream community dynamics, with their activity reflecting that of their hosts.

## Methods

### Study site, experimental design, and sample collection

Flume mesocosms were installed next to the *Dranse de Ferret*, a glacier-fed stream (GFS) in the Val Ferret catchment (Swiss Alps; 45.906 N, 7.124 E; 1774 m above sea level), during summer 2022. Specifically, streamwater was pumped from the GFS into three replicate channels (length 250 cm; width 3 cm) constructed of polyvinyl chloride (PVC) walls (height: 5 cm) mounted on a wooden surface, and protected from rain by a transparent PVC roof. Sterile clay coupons (2 × 2 × 0.33 cm) as substratum for biofilm, as used in previous studies [27, 29], were embedded in the channels allowing biofilm growth. To avoid heavy siltation on the biofilms, the GFS water was first pumped into two subsequent sedimentation tanks before being transferred to a header tank [27]. The discharge across channels simulated a natural flow regime downscaling The 2018 hydrological year of *Dranse de Ferret* (based on the Metabolism in Alpine Streams project; https://metalp.epfl) and was controlled via ball valves [27]. To simulate drought conditions, we manipulated the flow to include three successive zero-flow periods (D1: lasting 6 h on day 24; D2: 24 h on day 38; D3: 24 h on day 87). This corresponds to the C_temp_-I_flow_ treatment (ambient temperature and intermittent flow) of the experimental design represented in Touchette et al. (2025) [27]. Biofilms were collected from the coupons every 2–5 days from June 15th to July 27th, subsequently every 7 days until August 31 st, and again every 2–5 days until September 21st. This resulted in 22 sampling days. At each sampling, triplicate coupons (one per replicate channel) were collected for biofilm DNA extraction and chlorophyll-*a* quantification, immediately flash-frozen on dry ice, and stored at – 80 ºC and – 20 ºC, respectively, until processing. In addition, at sampling days before and after each drought, triplicate coupons (one per channel) for RNA/protein extraction were collected by submersion in 5 ml of DNA/RNA Shield (Zymo Research) prior to being mixed and flash-frozen on dry ice. Samples were stored at − 20 ºC until analysis. All sampling devices were flame-sterilized before use. Biomass of phototrophs was estimated by quantifying biofilm’s chlorophyll-a concentration via ethanol extraction, as described by Touchette et al. (2025).

### DNA extraction and shotgun metagenomic analysis

Biofilms were scraped from coupons in ice-cold molecular-grade water to create a slurry and were processed for DNA extraction using an optimized phenol–chloroform protocol [30], with the addition of a homogenization step by bead beating at 6000 r/m (Pre-cellys, Bertin Technologies), with a 2 × 15 s and a 15 s break program in 1.5 ml tubes containing 10–20% of 0.1 mm zirconium beads [27]. Short-read shotgun metagenomic libraries were performed using the NEBNext Ultra II FS library kit (New England Biolabs), following the manufacturer’s protocol. The protocol involved a 5-min fragmentation of 50 ng DNA stored in TE buffer (equal ratio of each triplicate pool together), followed by 6 PCR amplification cycles. This resulted in 22 gDNA libraries, where the size of ~ 500 bp was confirmed on a TapeStation D1000 ScreenTape (Agilent), and the concentration was quantified using a Qubit 1 × dsDNA HS kit (Invitrogen). A negative control was also added, following the exact same procedure, but using sterile TE buffer as input material. Libraries were pooled and sequenced on the AVITI platform with 300 cycles in paired-end configuration (Element Biosciences) at the EPFL Gene Expression Core Facility. Long-read shotgun metagenomic libraries were performed with the ligation sequencing gDNA Native Barcoding kit 24 V14 (Oxford Nanopore), following the manufacturer’s protocol, but using the Quick T4 DNA ligase and NEBNext Quick ligation reaction buffer (New England Biolabs) for both ligation steps. For input, extracted DNA samples were removed of smaller DNA fragments by bead selection, with a ratio of 0.6X SPRI beads (Beckman Coulter). Libraries were sequenced on a minION Mk1B using R10.4.1 flow cells (Oxford Nanopore).

Short-read metagenomic sequence data were processed as described by Michoud et al. (2025), and available on GitHub (https://github.com/michoug/VanishingGlacierMAGs). In brief, sequences were quality-based filtered and trimmed from adapters with *TrimGalore* v.0.6.10 [31] and human contamination was removed with *strobealign* v.0.14 [32] and *samtools* v.1.9 [33] against the human genome (GCF_000001405.40). Resulting reads were co-assembled with *MEGAHIT* v.1.2.9 with default parameters [34], using the 22 samples. Due to potential strain-level differences, we also assembled reads with subsets of samples, resulting in 5 co-assemblies of 4–5 subsequent samples, and two co-assemblies performed with the samples before or after the second drought period. Reads’ taxonomy was assigned with *SingleM* v.0.18.3 [35]. Long read metagenomic raw sequence data was basecalled, demultiplexed, and trimmed using *dorado* v.0.8.0 (ONT) in “duplex sup” mode and were assembled using *metaFlye* v.2.9.5 [36]. All assemblies were binned with *CONCOCT* v.1.1.0 [37], *COMEBin* v.1.0.3 [38], *MetaBAT2* v.2.15 [39], *MetaBinner* v.1.4.3 [40], *Rosella* v.0.5.4 [41], *SemiBin* v.2.1 [42], and *Vamb* v.4.1.3 [43]. Non-redundant set of bins per sample were generated using *DAS Tool* v.1.1.7 [44], and refined using *Rosella*. The resulting bacterial bins were dereplicated with *Galah* v.0.4 [45], their quality and completeness assessed with *CheckM2* v.1.0.2 [46], and their taxonomy identified with *GTDB-Tk* v.2.2.2 [47]. Bacterial MAGs with a medium-to-high quality threshold (> 70% completeness and < 10% contamination) were kept and functionally annotated with *Bakta* v.1.10.3 [48] and *eggNOG-mapper* v.2.1.12 [49]. A phylogenetic tree of bacterial MAGs was generated with *FastTree* v.2.2.11 [50] based on the alignment of 120 genes obtained from *GTDB-Tk*.

Refined bins more than 2.5 Mbp in size were analysed with *Whokaryote* v.1.1.2 [51], of which bins with more than 80% eukaryotic contigs were kept and considered as eukaryotic bins. *BUSCO* v.5.8.0 [52] was used to estimate their quality, and bins with a completeness > 30% were considered as eukaryotic MAGs and were dereplicated with *Galah*. Resulting medium-to-high quality MAGs were assigned taxonomy with *PhyloFisher* v.1.2.14 [53], and annotated with *MetaEuk* v.7.bba0d80 [54] and *eggNOG-mapper*. DNA coverage of bacterial and eukaryotic MAGs was estimated with *CoverM* v.0.7.0 [55], using *minimap2* [56], with the “trimmed_mean” method after filtering out reads < 90% identity and > 70% aligned fraction.

### RNA extraction and metatranscriptomic analysis

Biofilm samples for RNA extraction were defrosted while mixed on a rotator mixer to allow complete biofilm detachment from coupons. Coupons were removed from tubes using forceps. Samples were further mixed by pipetting, and 250 µl were transferred to 1.5 ml tubes and frozen at −20ºC until protein extraction (see Section "Protein extraction, mass spectrometry, and protein sequence analysis"). From the remaining slurries, 4 ml were transferred into Precellys Glass kit 0.1/0.5 mm 7 ml tubes (Bertin Technologies), and samples were lysed by bead beating on a Precellys Evolution Touch (Bertin Technologies) at 6500 r/m with a 2 × 25 s and 30 s break program. Lysed samples were centrifuged at 4500 × *g* for 1 min to settle beads, transferred into a new tube, and centrifuged again at 16,000×*g *for 1 min to pellet fine sediment particles. Clear supernatants were further lysed by mixing in an equal volume of TRI Reagent (Zymo Research).

RNA was purified with the Direct-zol RNA Miniprep kit (Zymo Research) including the DNAse treatment, according to the manufacturer’s protocol, with the exception that a vacuum pump was used to pass the mixture into the Zymo-Spin IICR column. RNA was further purified using the RNA Clean & Concentrator-5 (Zymo Research), again including the DNAse treatment step. RNA and DNA concentrations were assessed with the Qubit RNA HS and Qubit dsDNA HS kits, respectively. The absence of DNA in the RNA samples was further confirmed by a PCR targeting 16S rRNA genes run on TapeStation D1000 ScreenTape (Agilent). To confirm the RNA integrity, samples were run on a TapeStation High Sensitivity RNA ScreenTape, where a RIN ~ 6 was achieved. A negative control was also added, following the exact same procedure, but using sterile DNA/RNA Shield as input material. 

Total RNA was converted to cDNA, depleted from rRNA, and assembled into libraries using the Zymo-Seq RiboFree Total RNA Library Kit (Zymo Research). This was performed according to the manufacturer’s protocol, using the suggestions for low concentration input: (i) 1:4 dilution of the cDNA Synthesis Reagent 1; (ii) 90 min of depletion; (iii) 12 PCR amplification cycles. This resulted in 19 mRNA libraries (18 samples and one negative control), where libraries showed a size of ~ 450 bp on a TapeStation D1000 ScreenTape, with no detectable product in the negative control. Libraries were pooled and sequenced on an AVITI platform with 300 cycles in paired-end configuration at the EPFL Gene Expression Core Facility. RNA extraction was followed immediately by cDNA conversion without any freeze–thaw cycles to avoid RNA degradation. For all elution steps, an extra minute incubation on the column was added. All material used was sterile and free of RNAse/DNAse.

Raw transcript sequences were quality-based filtered and trimmed from adapters with *TrimGalore*, using a minimum length of 15 nt, but other default parameters. To ensure complete absence of rRNA sequences, trimmed transcripts were sorted with *SortMeRNA* v.4.3.7 [57]. Paired mRNA sequences were aligned to the 125 medium-to-high quality MAGs’ coding sequences using *Kallisto* v.0.51.1 [58] with 99 bootstraps, providing mRNA transcript abundance quantification for downstream differential abundance analyses. All gene sequences in the negative control were removed from other samples. RNA coverage of bacterial and eukaryotic MAGs was estimated with *CoverM* using the parameters described above. Taxonomy was also assigned to all transcripts using *SingleM.*

### Viral metagenome and metatranscriptome assembled genomes

The short reads co-assembly from all metagenomes and a co-assembly from all metatranscriptomes, performed with *MEGAHIT*, were used to identify both DNA and RNA-derived viral contigs. Viral contigs in the metagenome and metatranscriptome assemblies were identified with *VIBRANT* v.1.2.1 [59] and *geNomad* v.1.11.0 [60], and only viral contigs longer than 5 kbp were kept as viral MAGs (vMAGs). vMAG quality was assessed with *CheckV* v.1.0.3 [61]. Taxonomy was assigned using *geNomad* and complemented with “hmmsearch” from *HMMER* v.3.4 [62] to identify long terminal repeat (LTR) retrotransposons via reverse transcriptase domains (PF00078). Putative microbial hosts were predicted with *iPHoP* v.1.4.1 [63] for DNA vMAGs and *RNAVirHost* v.1.0.5 [64] for RNA vMAGs. Paired mRNA sequences were aligned to all vMAGs using *Kallisto*, and coverage was estimated with *CoverM*, as described above.

### Protein extraction, mass spectrometry, and protein sequence analysis

To extract GFS biofilm proteins, samples were mixed 2:1 in lysis buffer (10 mM DTT, 50 mM Tris–HCl (pH = 8), 0.1% Tergitol, 4% SDS), and bead-beaded at 6000 r/m, with a 3 × 1 min and 5 min pauses, on a Precellys Evolution Touch, using 10 mm glass beads (< 106 µm; Sigma-Aldrich). Samples were centrifuged at 16,000 g for 15 min at 4 ºC, and lysates were denatured according to the Blue Protein Loading Dye protocol (New England Biolabs), with a 5 min incubation at 99 ºC. Subsequently, 50 µl were run for 10 min at 270 V in a Mini-PROTEAN TGX precast gel (Bio-Rad Laboratories), followed by staining with QuickBlue Protein Stain (Lubio Science) for 1 h. Gels were destained overnight in deionized water and cut into 1 × 1 mm pieces. At the EPFL Proteomics Core Facility, gel pieces were washed twice with 50% ethanol in 50 mM ammonium bicarbonate (AB, Sigma-Aldrich) for 20 min and dried by vacuum centrifugation. Proteins were reduced with 10 mM dithioerythritol (Merck-Millipore) for 1 h at 56 °C, followed by similar washing and drying. Alkylation was performed with 55 mM iodoacetamide (Sigma-Aldrich) for 45 min at 37 °C in the dark, followed by similar washing and drying. Proteins were digested overnight at 37 °C with 12.5 ng/µl trypsin (Trypsin Gold, Promega) in 50 mM AB + 10 mM CaCl₂. Peptides were extracted twice for 20 min in 70% ethanol, 5% formic acid (FA, Merck-Millipore), desalted on C18 StageTips, and detergent was removed with the HiPPRTM kit (Thermo Fisher), following the manufacturer’s protocol. Resulting purified peptides were desalted and dried as described above. Mass spectrometry (MS) was performed as described by Sun et al. [65], but with separation conducted on a Vanquish Neo nano UPLC system on-line connected with an Orbitrap Fusion Lumos Tribrid Mass Spectrometer (Thermo Fischer Scientific).

Protein sequences were analysed using a pipeline available on GitHub (https://github.com/MACE-laboratory/MACE_metaproteomics). In brief, raw mass spectrometry files were converted with *ThermoRawFileParser* v.1.4.5 [66], and Label-Free Quantification (LFQ) was performed with *Sage* v.0.14.6 [67]. Protein intensities were identified with *Picked Group FDR* v.0.9.0 [68], using a database constituted of all protein sequences originating from our microbial MAGs, and of potential contaminants from human handling and trypsin digestion.

### Data analysis and statistics

Differences in phyla’s transcript relative abundance (before versus after droughts) was assessed with the Kruskal–Wallis Rank Sum Test, after confirmed non-normality with the Shapiro–Wilk test, both from the R package *stats* v.4.4.2 [69], and effect size was computed with “wilcox_effsize” function from *rstatix* v.0.7.1 [70]. Microbial community composition was assessed with Bray–Curtis dissimilarity on Hellinger-transformed data, using the “vegdist” and “transform” functions of *vegan* v.2.6–10 [71] and *microbiome* v.1.28.0 [72] R packages, respectively. Change in composition was explored with a nonmetric multidimensional scaling (NMDS), using “metaMDS” of *vegan* with 100 iterations. The effect of succession time and drought periods was assessed by fitting the NMDS scores with the “envfit” function and on the overall microbial composition with the “adonis2” function, both from *vegan* and with 999 permutations.

Differential abundance analyses were performed for each drought independently with *DESeq2* v.1.46.0 [73] to test the effect of droughts on gene expression and MAGs activity. For each drought, estimated counts from the *Kallisto* output were filtered to keep only transcripts present in at least two samples and in at least ten copies, and using three different approaches: (i) global scaling method, testing which gene transcripts are differentially abundant in the overall MAG community; (ii) taxon-specific scaling method, testing which gene transcripts are differentially abundant within each MAG, following a modified *DESeq2*-based pipeline from [74]; (iii) MAG differential activity method, testing their total transcript differential abundance (i.e., their activity), where estimated counts of each MAG were summed. Gene expression differences between samples were explored with a principal component analysis ordination (PCA) on variance-stabilizing transformed data from the *DESeq2* “vst” function, after running *DESeq2* on the entire dataset. Only gene transcripts or MAGs with a Benjamini–Hochberg adjusted *p*-value < 0.05 and a log_2_ fold-change (log_2_FC) ≥|2.0| were considered as significantly different. Differential abundance analysis was also performed on DNA and RNA viral MAGs using *DESeq2*, with the same design and statistical thresholds.

Significantly different genes from both global and taxon-specific scaling *DESeq2* results were assigned function with KEGG Orthology (KO) database, based on *eggNOG-mapper* annotations. Annotations of “Organismal Systems”, and “Human Diseases” categories and of “Poorly characterized” subcategory were removed. Moreover, functional assignment of prokaryotic genes to the subcategories “Transport and catabolism”, “Cell growth and death” (excluding the “Cell cycle–Caulobacter” pathway), and to the “Ubiquitin mediated proteolysis” pathway was also removed. The resulting lists of significant KO functions were assigned to the taxonomic groups *Stramenopiles*, *Cyanobacteriota* and “heterotrophs” and implemented in the “enrichKEGG” function of the *clusterProfiler* v.4.14.6 R package [75], to perform pathway enrichment analyses. The relative MAG-specific activity and significant function-specific differences pre- and post-drought were also visualized with heatmaps using normalized transcript per million (TPM), after filtering for transcripts with at least 0.1 TPM in at least two samples. Similar expression patterns were clustered by similarity in heatmaps, using Euclidian distance implemented with the “vegdist” and “hclust” functions of *vegan* and *stats*, respectively. Only identified protein with a false discovery rate (FDR) < 0.05 were kept and protein identified as potential contaminant or reverse sequences were removed. Protein functions were assigned using PFAMs from *eggNOG-mapper* annotations, as described above.

The ratio of phototrophs was estimated by dividing the sum of phototrophic eukaryotic reads by the sum of cyanobacterial reads, per metagenomic sample (*SingleM*). *Cryptophyceae*, *Glaucocystophyceae*, Haptista, *Rhodophyta*, Sar, and *Viridiplantae* were considered as phototrophic eukaryotic phyla. Shared phototrophic functions between diatom and cyanobacterial MAGs were assessed by uploading unique KOs derived from each taxonomical group to *KEGG Mapper—Reconstruct* [76], where the “Photosynthesis”, “Photosynthesis–antenna proteins”, and “Carbon fixation by Calvin cycle” were investigated. Data visualization was performed using the R packages *phyloseq* v.1.50.0 [77], *ggplot2* v.3.5 [78], *ggtree* v.3.14.0 [79] and *microViz* v.0.12.6 (Barnett et al*.* 2021).

## Results

### Multi-domain microbiome of glacier-fed stream biofilms

Metagenomic sequencing yielded an average of 20,348,534 ± 3,261,103 short reads and 484,987 ± 279,100 long reads of good quality per sample (Supplementary Table S1). Co-assemblies (short reads: max 1,085,346 contigs, long reads: 116,784 contigs; Supplementary Table S2) generated 2407 bins, of which 125 MAGs were classified as medium-to-high quality, including 117 bacterial MAGs (bMAGs) and 8 eukaryotic MAGs (eMAGs). These MAGs recruited, on average, 21.0 ± 3.5% of metagenomic reads per sample.

**Fig. 1:**
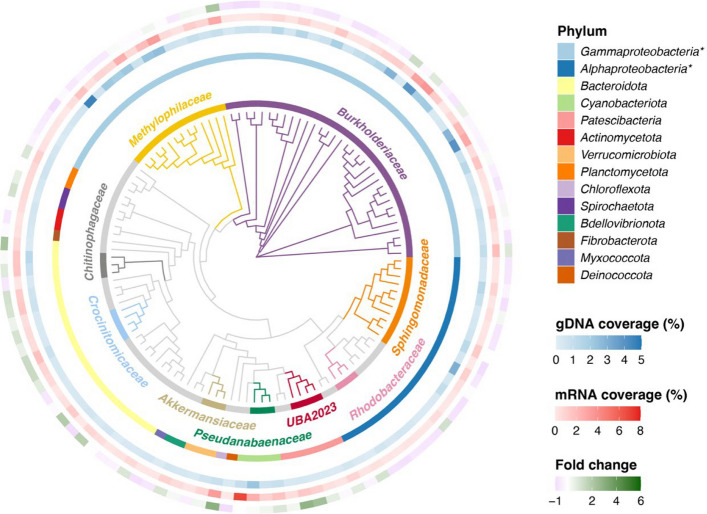
Bacterial MAGs recovered from a glacier-fed stream biofilm growth experiment under repetitive droughts. Maximum likelihood phylogenetic tree of bacterial MAGs. The branches and inner ring colors correspond to the taxonomy of bacterial MAGs at the family level. The second ring colors indicate the taxonomy at the phylum level. The blue gradient color ring shows the relative metagenomic coverage (gDNA) of each MAG. The red gradient color ring shows the relative metatranscriptomic coverage (mRNA) of each MAG. The ring with a gradient color from pink to green represents the fold change between gDNA and mRNA. A green color indicates relatively more transcripts than genomic material, meaning an overrepresentation of the MAG in the metatranscriptomes. * Gammaproteobacteria and Alphaproteobacteria are classes of the Pseudomonadota phylum

bMAGs accounted for 96.5% of the total relative abundance of the 125 MAGs, while eMAGs represented only 3.5%. This corresponds to an underrepresentation of the Eukaryota, especially phototrophs, and Archaea domains in our samples, as they accounted for 23.7 ± 10.8% and 2.5 ± 0.2% of the total metagenomic reads, respectively (Fig. S3). bMAGs varied in size (0.59–8.86 Mbp) and GC content (30–68%) (Fig. S1, Supplementary file S1). All bMAGs were taxonomically classified to the genus level, except 10, which were assigned only to the family level. bMAGs were dominated by the Pseudomonadota (*Gammaproteobacteria*, *n* = 50, 60.5% bMAGs relative abundance; *Alphaproteobacteria*, *n* = 21, 13.1%) and Bacteroidota (*n* = 21, 9.4%) phyla, and the *Burkholderiaceae* (*n* = 33, 38.9%), *Methylophilaceae* (*n* = 13, 20.2%), and *Sphingomonadaceae* (*n* = 11, 9.3%) families (Fig. 1). Seven out of the eight eMAGs (Supplementary file 2) were from the *Stramenopiles* clade, part of the *Ochrophyta–Bacillariophyta* phylum (diatoms) (Fig. S2), representing 3.3% of the MAGs community relative abundance. These dominant bacterial and eukaryotic taxa also dominated the overall metagenomes (Fig. S3, Fig. S4). One eMAG is from the *Fungi* kingdom.

(*Obazoa*) was also generated (Fig. S2), closest to the *Paramicrosporidium* genus.

Exploring the beta-diversity of the MAG community, we found that ecological primary succession significantly influenced community composition, showing a strong and significant association (“envfit”, *p* = 0.001) with the first NMDS axis (Fig. S4). Additionally, the MAGs’ community composition varied significantly based on drought periods (“envfit”, *p* = 0.001), as indicated by increased dissimilarity along the second NMDS axis following the second drought event (D2, day 38) (Fig. S4). This pattern was further supported by PERMANOVA (*R*^2^ = 0.8, *p* = 0.001).

Metagenomic and transcriptomic analyses also generated viral MAGs (vMAGs), resulting in 2242 DNA-derived vMAGs and 333 RNA-derived vMAGs (Supplementary file 3). However, when considering only high-quality vMAGs (> 95% completeness), we obtained 99 DNA-derived vMAGs and 127 RNA-derived vMAGs. All vMAGs ranged between 1.2% and 8.8% of metagenomic reads and between 2.0% and 32.3% of metatranscriptomic reads per sample. The most abundant DNA vMAGs were from the *Caudoviricetes* (63.0 ± 22.6%) and *Arfiviricetes* (18.1 ± 24.3%), *Megaviricetes* (4.6 ± 2.9%) classes (Fig. S5). Up to 10.0 ± 5.3% of the DNA virus community was composed of LTR retrotransposons (Fig. S5). The DNA-derived vMAG community also exhibited a strong successional pattern, with a substantial increase in *Arfiviricetes* towards the end, and a spike in virus abundance before the third drought (Fig. S5). The RNA-derived viral community was dominated by RNA viruses of the *Chrymotiviricetes* (66.4 ± 12.0%), *Alsuviricetes* (10.7 ± 4.7%), and *Pisoniviricetes* (5.0 ± 5.0%) classes (Fig. S5). In a minor proportion, transcriptomic reads also generated DNA viruses, including *Caudoviricetes* (4.7 ± 7.5%), *Arfiviricetes* (3.8 ± 8.5%), and *Megaviricetes* (0.05 ± 0.09%), but also LTR retrotransposons (0.06 ± 0.1%), most likely from their transcripts (Fig. S5), indicating that these DNA viruses were actively infecting.

### Drought impacts on microbial activity

**Fig. 2:**
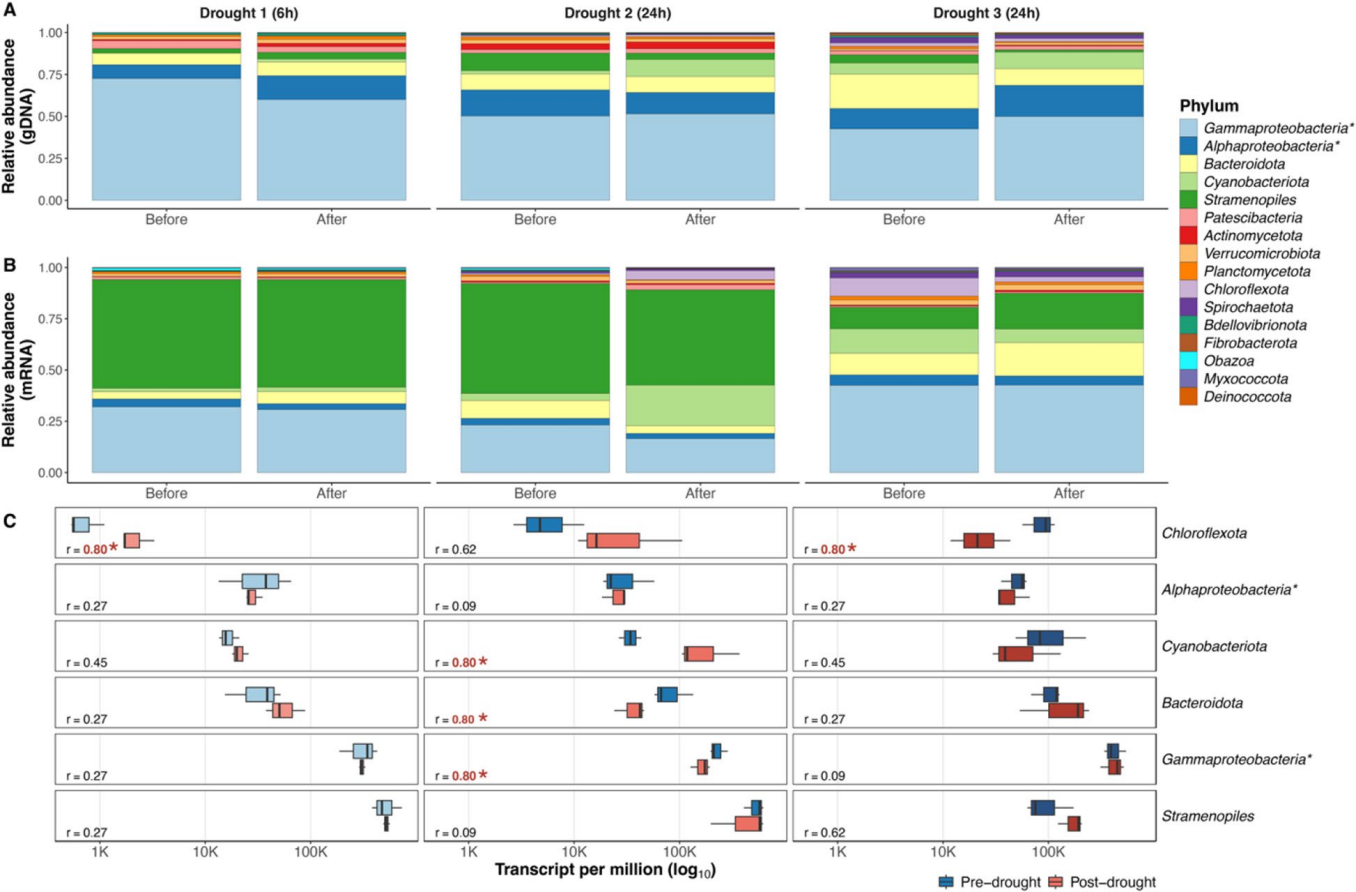
Drought-induced changes in MAGs’ composition (metagenome) and activity (metatranscriptome) of glacier-fed stream biofilms. **A** Relative abundance of the bacterial and eukaryotic MAGs within each pre- and post-drought metagenome. **B** Relative abundance of transcripts (i.e., activity) assigned to MAGs within each pre- and post-drought metatranscriptome. Metatranscriptomic replicate samples were merged for visualization. **C** Differences in relative activity between pre- and post-drought conditions for each drought period, for the most abundant MAG taxonomic groups. “r” is the standardized effect size between the two conditions. Bolded red text with a star (*) indicates a significant difference (*p* < 0.05) in relative activity between pre- and post-drought conditions for each drought period. * Gammaproteobacteria and Alphaproteobacteria are classes of the Pseudomonadota phylum

Metatranscriptomic sequencing generated 32,766,859 ± 17,291,754 quality reads, resulting in 7,991,363 ± 4,453,828 mRNA transcripts on average per sample (Supplementary Table S3). Additionally, we recovered 22 proteins in total, all originating from eMAGs (Supplementary file S4). Unfortunately, we did not identify proteins originating from bacterial and viral MAGs, likely due to the technical challenges of extracting proteins from complex glacier-fed stream biofilms, which are typically rich in EPS and mineral particles but low in biomass. As protein identification was technically limiting, we used these data only to support broad patterns observed in the metatranscriptomic results of diatom MAGs. A more in-depth metaproteomic analysis would be required to assess the translational activity of other biofilm members. When quantifying the expression of the MAGs’ coding sequences (i.e., transcriptomic activity) using TPM, 60.6 ± 21.8% of mRNA was attributed to bMAGs, and 39.4 ± 21.8% to eMAGs (Fig. 1, Fig. S2). Based on transcript abundance, the most active MAGs were those affiliated with diatom *Stramenopiles* (38.9 ± 21.6% of mapped transcripts), *Gammaproteobacteria* (31.3 ± 12.0%), *Bacteroidota* (8.1 ± 6.0%), and *Cyanobacteriota* (7.6 ± 9.2%) (Fig. 2, Fig. S6). Overall, this MAG-derived activity profile resembled that of the entire metatranscriptomes; however, the metatranscriptomes also contained a large proportion of transcripts from Metazoa and Fungi (non-phototrophic eukaryotes, Fig. S7), which are not the focus of this study. Moreover, the overall high activity of diatoms was corroborated by the consistent recovery of their housekeeping proteins (ATPase, actin, histone, RAD51) across replicates, thus indicating active translational activity (Supplementary file S4).

**Fig. 3:**
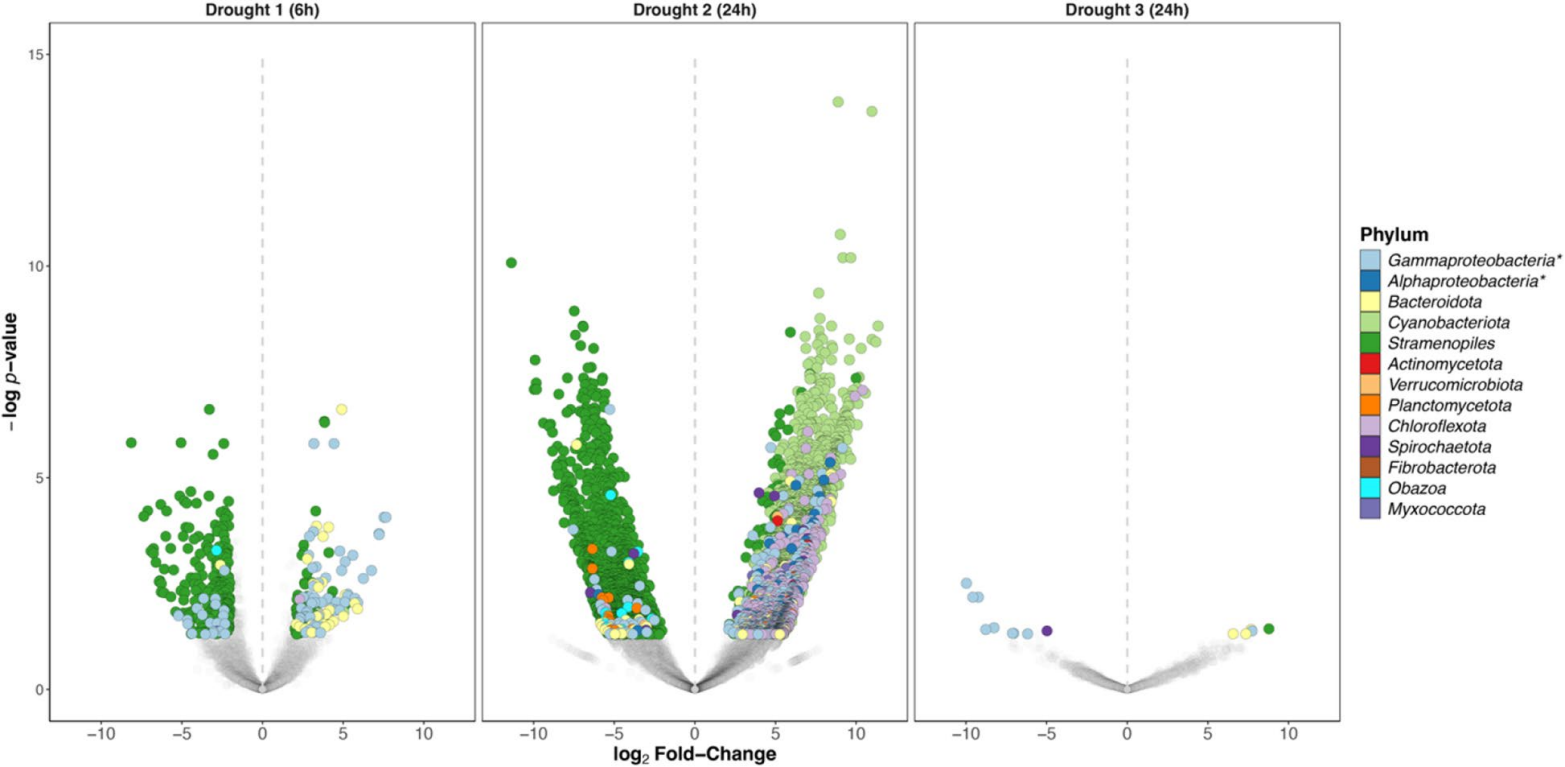
Differential abundance analyses showing the global-scaled differentially expressed genes between pre- and post-drought samples. Global-scaled drought-induced significant differentially expressed genes (DEGs; *p*-value < 0.05, log2 fold change higher than 2 or lower than − 2), where positive log2 fold-change indicates up-regulated genes after a drought event (right of the dashed line), and negative log2 fold-change indicates down-regulated genes after a drought event (left of the dashed line). DEGs are color-coded by taxonomy of their MAG of origin (phylum level). * Gammaproteobacteria and Alphaproteobacteria are classes of the Pseudomonadota phylum

Analysing the effect of droughts on the relative transcriptional activity of each phylum, no obvious pattern was consistent between the three drought periods. The second drought impacted the MAGs’ activity the most, particularly by reducing the relative transcriptional level of most bMAGs, but by increasing the relative transcriptional level of *Cyanobacteriota* (Fig. 2) and *Patescibacteria* (Fig. S8). Principal component analysis (PCA) of variance-stabilized expression data revealed clear clustering of the metatranscriptomic replicate samples, with separation between pre- and post-drought samples observed for the first two drought periods (Fig. S9). Global-scaled differential expression analysis between pre- and post-drought samples (community-wide response), performed independently for each drought, identified 8271 differentially expressed genes in total (D1: 137 up, 270 down, 407 total; D2: 5783 up, 2,067 down, 7850 total; D3: 5 up, 9 down, 14 total; Fig. 3). Across droughts, only 97 genes were shared between D1 and D2: 57 were consistently down-regulated, 4 consistently up-regulated, and 36 showed contrasting expression patterns. Following the 6 h drought, 91.1% of down-regulated genes were from *Stramenopiles* (246 genes), while up-regulated genes were mostly associated with *Gammaproteobacteria* (40.9%, 56 genes), *Stramenopiles* (37.2%, 51 genes), and *Bacteroidota* (19.7%, 27 genes) (Fig. 3). Similarly, 96.0% of down-regulated genes after the second drought were from *Stramenopiles* (1985 genes), but up-regulated genes were in the majority from *Cyanobacteriota* (72.0%, 4169 genes) (Fig. 3). Relatively few transcriptional changes were identified following the third drought (Fig. 3). Taxon-scaled differential analysis between pre- and post-drought samples (MAG-specific response) identified 4392 differentially expressed genes in total (D1: 150 up, 246 down, 396 total; D2: 2604 up, 1384 down, 3988 total; D3: 3 up, 5 down, 8 total; Fig. S10). All of these genes were unique to a specific drought, and most of them (3988) originated from the second drought period and occurred in *Stramenopiles* (70.8%, 3109 genes) (Fig. S10).

**Fig. 4:**
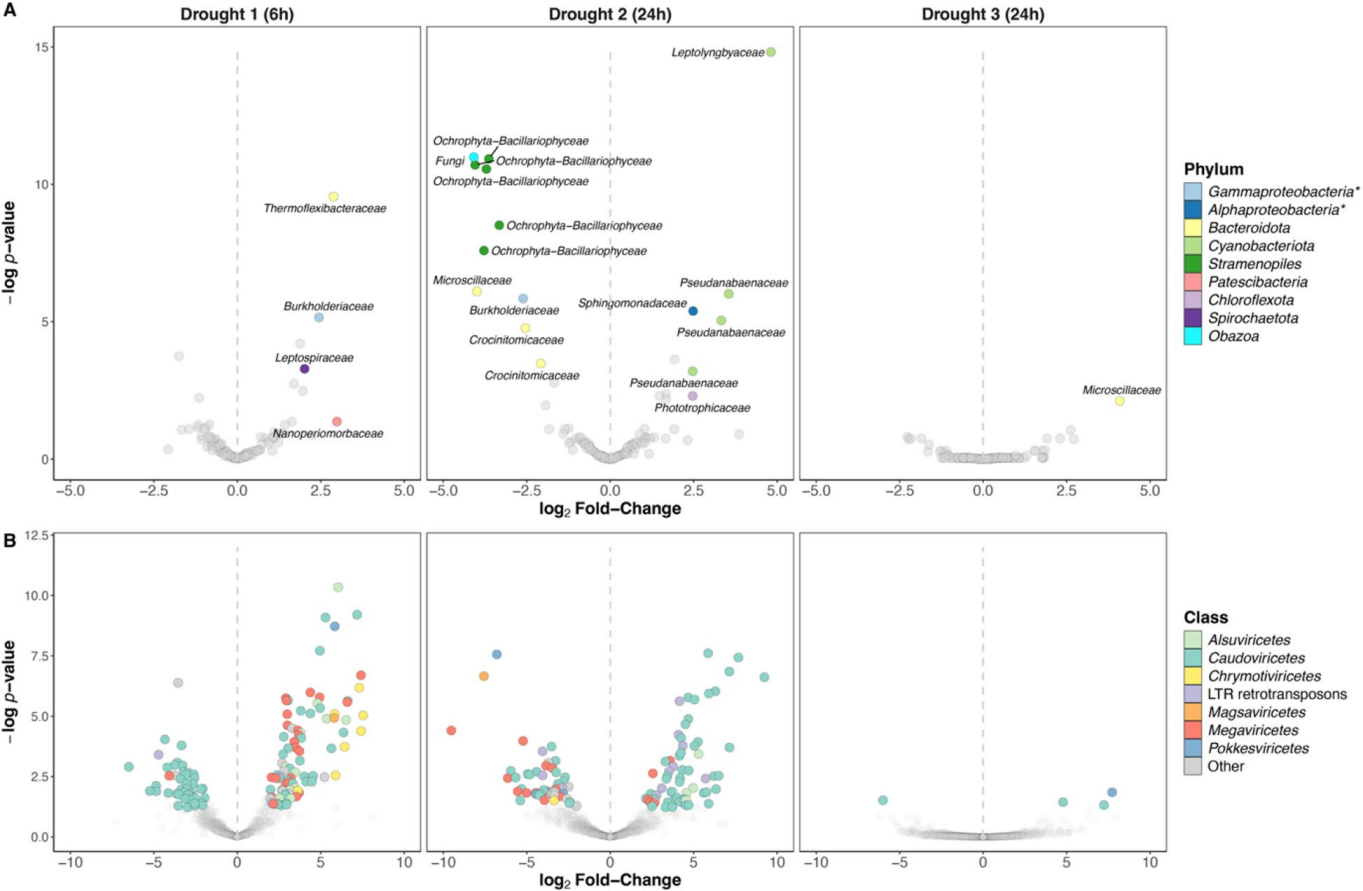
Differential abundance analyses showing MAG-specific differential activity between pre- and post-drought samples. **A** Drought-induced differentially active microbial MAGs. **B **Drought-induced differentially active viral MAGs. Positive log2 fold-change indicates up-regulated MAG after a drought event (right of the dashed line), and negative log2 fold-change indicates down-regulated MAG after a drought event (left of the dashed line). * Gammaproteobacteria and Alphaproteobacteria are classes of the Pseudomonadota phylum

A differential transcriptional activity analysis of each MAG was then performed by summing all transcripts associated with each MAG. Overall, we found that only four bMAGs had an increased activity following the first drought, and only one bMAG for the third drought, while none had reduced activity (Fig. 4). The second drought had a more pronounced effect on transcriptional activity. It was responsible for a decrease in the activity of most eMAGs, including 5 out of 7 algae and the unique fungus, as well as 4 bMAGs. This drought also stimulated 6 bMAGs, including all 4 *Cyanobacteriota* and our unique *Chloroflexota* MAG (Fig. 4, Fig. S11). In addition, 250 viruses were found to be differentially abundant following drought events (D1: 88 up, 45 down, 133 total; D2: 65 up, 48 down, 113 total; D3: 3 up, 1 down, 4 total; Fig. 4). While no clear taxonomic pattern was found across the three droughts, 25 *Megaviricetes* and 8 *Chrymotiviricetes* were more abundant after the first drought, while after the second drought, 8 virus-like LTR retrotransposons and 47 *Caudoviricetes* (including 4 *Kyanoviridae*) were upregulated. In accordance with previous results, the third drought induced little change in viral activity/abundance (Fig. 4).

### Drought impacts on metabolic functions

**Fig. 5:**
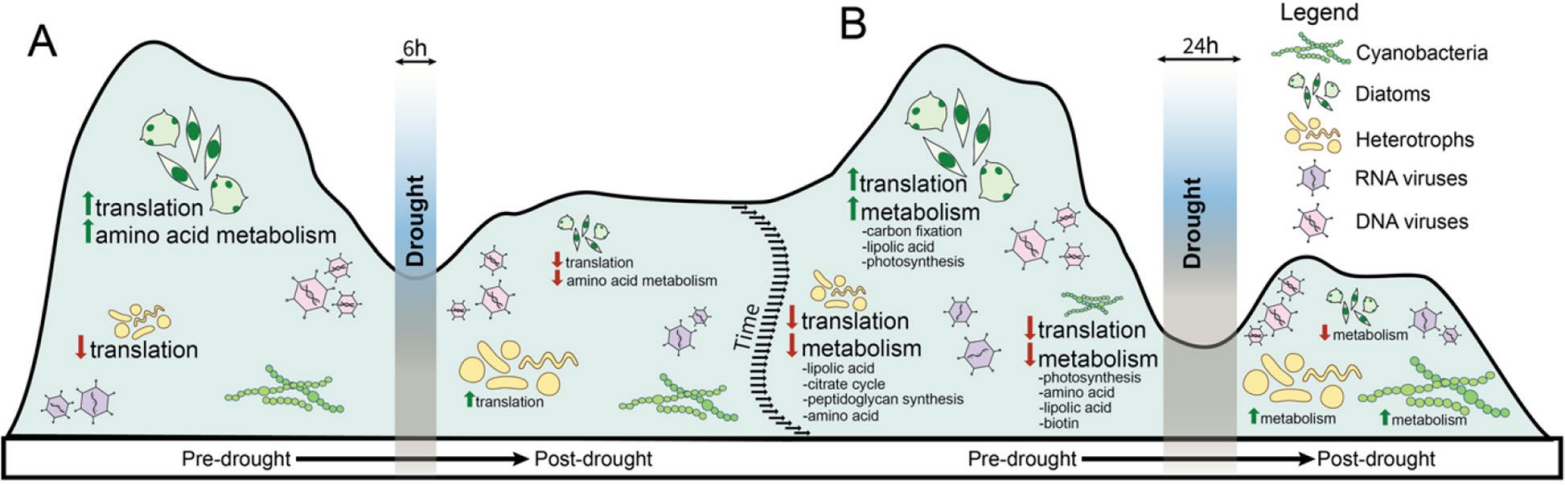
Schematic representation of drought-induced functional changes in the diatoms, cyanobacteria, and heterotrophs’ taxonomic groups within glacier-fed stream biofilms. **A** Drought-induced metabolic changes following the first drought (6 h). **B **Drought-induced metabolic changes following the second drought (24 h). For the second drought, only major functional changes are illustrated; a complete list is available in Supplementary file 4. Green upward arrows indicate functional pathway enrichment; red downward arrows indicate functional pathway suppression. The relative size of each illustrated taxonomic group reflects their relative activity change pre- and post-drought (not to scale)

On the functional level, The 8271 identified global-scaled differentially expressed genes were assigned to 1960 unique KEGG KOs, representing 287 different pathways within 31 subcategories (Fig. S12). By implementing these differentially expressed genes into enrichment analyses for each taxonomic group (i.e., diatoms, cyanobacteria, heterotrophs), the first drought was found to impact diatoms, by inducing a decrease in biosynthesis of amino acids and in ribosomal activity linked to translation, and to stimulate ribosomal activity in heterotrophs (Fig. 5, Supplementary file S5). The second drought induced strong changes in the metabolism: diatoms depleted 38 pathways, including translation, transcription, and overall metabolism (glycolysis, photosynthesis, nitrogen, etc.). In contrast, this drought enriched 32 pathways in cyanobacteria, and 57 in heterotrophs (Fig. 5, Supplementary file S5), especially towards biosynthesis of amino acids, carbohydrates, and energy metabolism. No functional changes were identified in the third drought (Fig. 5, Supplementary file S5). Using The 4392 taxon-scaled differentially expressed genes, enrichment analysis confirmed the upregulation of ribosomes and amino acid biosynthesis in diatoms following the first drought, and the absence of functional changes after the third drought (Supplementary file S6). However, taxon-scaled analysis identified a stimulation of glycerophospholipid metabolism in diatoms, and an enrichment of translation and amino acid biosynthesis metabolism in cyanobacteria, after the second drought (Supplementary file S6). Taxon-scaled analysis also suggests unchanged functionality in overall glacier-fed stream heterotrophs, following droughts. The overall major change in functionality associated with the second drought period was also visible in the change of relative expression of KEGG subcategories (Fig. S13) and pathways (Fig. S14).

**Fig. 6:**
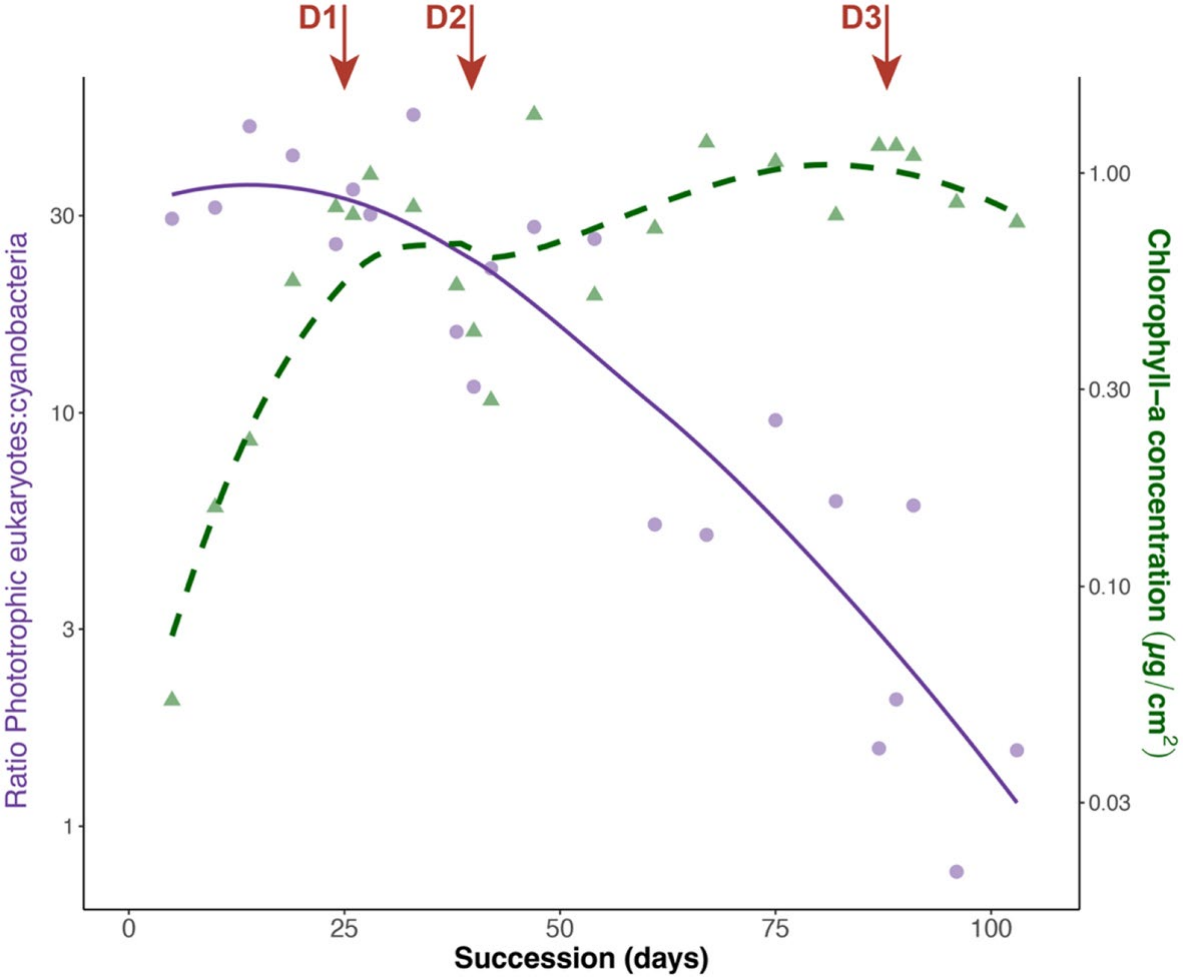
Functional redundancy in phototrophic members of glacier-fed stream biofilms. Evolution of chlorophyll-a concentration (phototroph biomass; green dashed line) and the ratio of phototrophic eukaryotes:cyanobacteria (sum reads phototrophic eukaryotes ÷ sum reads cyanobacteria; purple solid line) over the succession period. D1, D2, and D3 indicate the timing of the three droughts. *Y*-axes are shown on a logarithmic scale

Considering the apparent drought-induced shift in metabolic activity from diatoms to cyanobacteria, we compared phototrophic biomass (determined via chlorophyll-*a* concentration) to the abundance of phototrophic eukaryotes versus cyanobacteria, expressed as the phototrophic ratio. During succession, the phototrophic ratio decreased (Fig. 6), suggesting an increasing representation of cyanobacteria with time. Over the same period, phototrophic biomass initially increased rapidly, then reached a plateau with transient reductions following drought events (Fig. 6).

Diatoms and cyanobacterial MAGs shared all gene orthologs associated with the Calvin cycle (carbon fixation), except for the ribulose-1,5-bisphosphate carboxylase/oxygenase (RuBisCO), which was detected only in the cyanobacterial MAGs (Fig. S15). Major orthologs of the photosynthesis electron transport chain were also shared between both taxonomic groups (e.g., PETC, PETH, ATPF0a); however, several associated genes were detected exclusively in cyanobacteria (Fig. S16). The absence of key genes in diatom MAGs may be attributable to their lower genome completeness compared to cyanobacterial MAGs (Supplementary file S2), or result from technical challenges associated with recovering plastid genomes [80]. In contrast, antenna protein gene orthologs differed markedly between both groups: cyanobacteria encoded all genes associated with phycobilisome (allophycocyanin, phycocyanin, phycoerythrocyanin, and phycoerythrin), whereas diatoms encoded only genes associated with the light-harvesting chlorophyll protein complex (Fig. S17).

## Discussion

Despite the extreme conditions of glacier-fed streams, they host diverse and metabolically active biofilm communities [4, 5]. However, due to climate change, these biofilms will be increasingly exposed to more frequent and prolonged drought periods [15–17]. This study combines multi-omics analyses of glacier-fed stream biofilms to examine microbial activity and functionality following drought disturbances. We identified that droughts caused a shift in microbial phototrophs; specifically, diatoms were replaced by cyanobacteria following drought, while total biofilm chlorophyll-*a* remained overall stable, suggesting cross-domain functional redundancy. Additionally, upon rewetting, we observed metabolic stimulation of heterotrophic microorganisms and active transcription of DNA viruses within the biofilm community.

### Diatoms are highly active in glacier-fed stream biofilm

From our metagenome timeseries, we recovered MAGs across the Bacteria and Eukaryota domains, as well as DNA and RNA viruses. We acknowledge that these MAGs represent only a fraction of the total glacier-fed stream biofilm assemblage, yet they correspond to the principal taxonomic and transcriptionally active groups in the community (Fig. S3, Fig. S6). Congruently, the dominance of *Gammaproteobacteria*, *Alphaproteobacteria*, *Bacteroidota*, *Cyanobacteriota*, *Stramenopiles* (diatoms), and *Patescibacteria* in the MAG community (Fig. 1, Fig. S2, Fig. S4A) was consistent with previous reports from glacier-fed stream biofilms [5, 27, 81]. Importantly though, these taxa were not only present, but also transcriptionally active (Figs. 1 and 2), demonstrating their ecological success in glacier-fed streams as active members of the community. Over the course of the experiment, community composition changed strongly over time (Fig. S4B), reflecting the well-known successional turnover of freshwater biofilms [27, 82]. To avoid confounding drought effects with these underlying ecological successional dynamics, we analyzed microbial activity and functional responses to drought by comparing the pre-drought and post-drought samples of each drought event independently (see “Drought impacts phototrophic activity while promoting heterotrophic metabolism” section).

We identified RNA viruses in our glacier-fed stream biofilms, primarily from the *Chrymotiviricetes*, *Alsuviricetes*, and *Pisoniviricetes* classes (Fig. S5B), mostly predicted to infect fungi (Supplementary file 3). Although exploratory, these findings confirm for the first time the presence of RNA viruses in glacier-fed stream biofilms, further highlighting the complexity and understudied nature of their eukaryotic members. DNA viruses of the class *Caudoviricetes* (phylum *Uroviricota*) dominated our biofilm samples, and eukaryote-associated *Megaviricetes* were also detected (Fig. S5); both groups are typical viruses found in glacier-fed stream biofilms [5, 6]. However, the less common *Arfiviricetes* viruses emerged as dominant viruses during the succession, potentially reflecting a drought-induced compositional shift in the viral communities, as observed in other freshwater ecosystems [83].

Across the study, we identified diatoms (*Stramenopiles*) as the overall most transcriptionally active taxa in the biofilms (Fig. 2B). The discrepancy between an overall high transcriptomic activity and low genomic abundance of *Stramenopiles* (Fig. 2B, Fig. S2) implies that, although not the most abundant taxa, diatoms play a crucial functional role in glacier-fed stream biofilms. Concurrently, diatoms were also confirmed to be translationally active within the biofilm, as we identified proteins originating from *Stramenopiles* MAGs (Supplementary file S4). Phototrophs supply organic matter to heterotrophs through exudates [84]. This mechanism is thought to be an important source of organic carbon in the nutrient-limited glacier-fed stream environment [85]. Algae tend to occupy central positions in microbial glacier-fed stream networks based on OTU co-occurrences [86, 87]; although they are relatively low in abundance (Fig. 2A), their high activity (Fig. 2B) likely supports the biofilm microbial communities.

### Drought impacts phototrophic activity while promoting heterotrophic metabolism

Although diatoms were overall highly active, their relative importance largely decreased at later stages of succession (Fig. 2B), suggesting that drought events induce physiological stress in diatoms, which limits their recovery under repeated disturbances. This was further supported by the detection of the heat shock protein 90 (HSP90) originating from all six diatom MAGs (Supplementary file 4), implying active translation of these stress-associated proteins [88]. After the first drought, diatoms displayed early signs of metabolic stress, with the downregulation of translation and amino acid biosynthesis (Fig. 5). Moreover, this drought may have increased diatom vulnerability to viral infections, a stress-induced mechanism previously observed in marine diatoms and plants [89, 90]. This is supported by the pronounced drought-induced increase in the viral activity of *Megaviricetes* and *Chrymotiviricetes* (Fig. 4), which infect algae and algae-parasitizing fungi (chytrids), respectively. Therefore, drought stress may facilitate diatom infections by *Megaviricetes*, although this remains an inference solely based on putative host association. Similarly, given the high abundance of chytrids in glacier-fed streams [91] and the apparent drought-induced increase in *Chrymotiviricetes* activity, drought stress putatively increases chytrids’ parasitism of diatoms*.* The stress response was exacerbated after the second drought, where the activity of diatom MAGs and *Megaviricetes* was broadly suppressed (Figs. 3, 4). In addition, this drought upregulated eight LTR retrotransposons with *Viridiplantae* phyla (including diatoms) as the predicted host (Supplementary file 3), which have been suggested to play a role in diatom stress responses [92, 93]. Diatoms are recognized as ecological indicators of environmental stress and have been used to evaluate changes in water quality, lake acidification, and landscape disturbances [94–96], for instance. The clear drought-induced impacts on diatoms observed here are consistent with their well-documented environmental sensitivity and support that diatom communities may play a role as indicators of drought stress in glacier-fed streams, as used in low-land rivers [97].

Alongside the suppression of diatom activity, cyanobacterial MAGs were significantly upregulated following the second drought. This was evident in overall transcript abundance (Fig. 3), differential activity (Fig. 4), and functional gene expression (Fig. 5), suggesting a drought-induced metabolic shift from diatoms to cyanobacteria (Fig. 5). Furthermore, we observed increased activity of five *Caudoviricetes* viruses with cyanobacteria as their putative predicted hosts (Supplementary file 3), as well as of cyanophages from the *Kyanoviridae* family [98], suggesting a potential increase in viral infection associated with enhanced cyanobacterial activity. These patterns align with observations in an alpine river, where flow cessation caused a shift in total benthic chlorophyll-*a* contributions, mainly from diatoms to cyanobacteria [99], and support that diatoms are more sensitive to drought than cyanobacteria [100–102]. In contrast, cyanobacteria are well known for their desiccation tolerance [103] and their rapid growth capacity upon rewetting [104], hence the apparent resistance and metabolic stimulation following this 24-h drought event. Compared to diatoms, many cyanobacteria are mixotrophs [105], giving them an advantage in the fluctuating glacier-fed stream environment [5]. The metabolic shift from diatoms to cyanobacteria also coincides with the stimulation of heterotrophic transcriptomic activity (Figs. 3, 4), resulting in a global heterotrophic metabolism enhancement (Fig. 5). This may indicate an opportunistic metabolic activation upon rewetting, consistent with the rapid recovery of stream bacterial metabolism observed after droughts, as measured by enzymatic activity and oxygen production [100, 106, 107], mirroring the “birch effect” well documented in soils [108].

The clear pattern of heterotrophic metabolism enhancement and diatom stress observed during the two first droughts contrasted with the third drought, which did not result in noticeable differential activity and functional changes within the biofilm community (Figs. 3, 4). Although surprising, this observation aligns with the absence of detectable shifts in microbial community composition (Fig. S4), as previously reported under such perturbations [27]. Together, these results indicate that while longer droughts amplify their ecological impacts [22, 102], repeated drought exposure may select for glacier-fed stream biofilm communities more resistant to subsequent hydrological fluctuations [101].

### Glacier-fed stream biofilm phototrophs are functionally redundant

The lack of taxon-scaled metabolic enrichment in heterotrophs (Fig. 5), alongside the minimal changes in their transcriptomic activity (Fig. 4), points to the general resistance of biofilm bacteria and heterotrophic metabolism to moderate drought. This resistance likely reflects emergent properties of biofilms, such as desiccation tolerance [109], paralleling observations in non-GFS streams where CO_2_ fluxes and carbon assimilation were unaffected by droughts [22, 110]. In contrast, phototrophic organisms were more affected, but overall resilient to repetitive droughts, supported by only a transient decline of total phototrophic biomass (Fig. 6). This apparent resilience to drought was largely driven by the transcriptomic and functional stimulation of cyanobacteria (Figs. 4, 5), a pattern previously observed in freshwater ecosystems [22, 111]. Diatoms and other phototrophic eukaryotes, however, did not fully recover from the 24-h drought event, as indicated by their reduced relative abundance (Fig. S3, Fig. S4). Alongside the decreasing ratio of phototrophic eukaryotes:cyanobacteria during the experiment (Fig. 6), these results support a drought-induced shift in primary producers, favoring cyanobacteria over other phototrophs in glacier-fed stream biofilm. However, the overall similar total phototrophic biomass throughout the experiment (Fig. 6) suggests that phototrophic metabolism was maintained despite drought events, supporting the presence of functional redundancy in glacier-fed stream biofilms [5, 81]. This functional redundancy in phototrophy was further evidenced by diatom and cyanobacterial MAGs sharing many key gene orthologs in the photosynthetic electron transport chain and in the carbon fixation pathway (Supplementary Fig. S15–S16), even though they harvest light using different protein complexes (Supplementary Fig. S17).

Our results further suggest that glacier-fed stream heterotrophic bacteria are resistant to drought and that their metabolism is stimulated upon rewetting. In contrast, diatom activity was reduced by drought, although their abundance tended to recover over time, suggesting resilience to such environmental perturbation. Contrary to our hypothesis, we were not able to identify specific stress-response pathways. Instead, diatom activity appeared broadly suppressed, indicating a generalized downregulation rather than a targeted stress response. As expected, we observed potential functional redundancy of phototrophic metabolism, as phototroph biomass remained stable despite drought conditions. Finally, we detected transcriptionally active DNA and RNA viruses and observed changes in their activity in relation to host dynamics, thus suggesting that viral infections may potentially influence ecological dynamics [112] within the glacier-fed stream biofilms, particularly in response to stress. However, we do acknowledge that bioinformatic tools currently available for viral host prediction are not optimal, as this field is only starting to emerge, and emphasize that these interpretations should be considered as tentative and rather viewed as hypotheses than conclusive demonstrations of host-virus relationships. Nevertheless, by combining host prediction with viral taxonomy, our results suggest that mild drought stress may trigger *Megaviricetes’* infection in diatoms, indicating a “killing-the-losers” strategy, as observed in phytoplankton [113]. Additionally, as cyanobacteria become dominant within the biofilm, viruses targeting them seemed also stimulated.

## Conclusions

Our results suggest that glacier-fed stream biofilm microorganisms are generally resistant to mild drought events. However, prolonged drought stimulates their heterotrophic metabolism and induces a shift among phototrophic members, characterized by reduced activity of diatoms and increased abundance and activity of cyanobacteria. Signs of stress in diatoms further support this shift. The absence of noticeable impacts after the third drought suggests that once the glacier-fed stream biofilm microbiome is altered by drought, it remains stable and adapted to subsequent droughts. Overall, our study provides evidence of drought-induced changes in the composition, activity, and metabolic functioning of glacier-fed stream biofilm, with potential consequences for carbon cycling in high-mountain stream ecosystems.

## Supplementary Information

Supplementary Material 1: Table S1. Sequencing and processing statistics of the metagenomics samples. Table S2. Summary statistics of the metagenome (co)assemblies. Table S3. Sequencing and processing statistics of the metatranscriptomic samples.

Supplementary Material 2. File 1. General features and characteristics of the glacier-fed stream bacterial MAGs (CheckM2). File 2. General features and characteristics of the glacier-fed stream eukaryotic MAGs (BUSCO). File 3. General features and characteristics of the glacier-fed stream viral MAGs (CheckV). File 4. Results of protein identification from metaproteomic samples, and associated metadata. File 5. Results from the enrichment analysis (clusterProfiler) using the DEGs from global-scaling differential analysis, for each drought period. File 6. Results from the enrichment analysis (clusterProfiler) using the DEGs from taxon-scaling differential analysis, for each drought period.

Supplementary Material 3: Figure S1. Quality and characteristics summary of bacterial MAGs recovered from a glacier-fed stream biofilm growth experiment under repetitive droughts. Figure S2. Eukaryotic MAGs recovered from a glacier-fed stream biofilm growth experiment under repetitive droughts. Figure S3. Microbial composition of whole glacier-fed stream biofilm metagenomes. Figure S4. Successional patterns in glacier-fed stream biofilm microbiomes under repetitive droughts. Figure S5. Taxonomy and successional patterns of DNA and RNA viral MAGs in glacier-fed stream biofilm microbiomes under repetitive droughts. Figure S6. Drought-induced changes in MAG activity (metatranscriptome) of glacier-fed stream biofilms. Figure S7. Drought-induced changes in activity (whole metatranscriptome) in glacier-fed stream biofilms. Figure S8. Drought-induced changes in glacier-fed stream biofilm MAGs’ activity. Figure S9. Principal component analysis (PCA) plot of variance-stabilizing transformed counts, showing clustering of replicate samples. Figure S10. Differential abundance analyses showing the taxon-scaled differentially expressed genes between pre- and post-drought samples. Figure S11. Drought-induced relative changes in MAGs’ activity. Figure S12. Number of drought-induced DEGs per KEGG metabolic category for each drought period. Figure S13. Drought-induced relative changes in biofilm functionality (KEGG subcategories). Figure S14. Drought-induced relative changes in biofilm functionality (KEGG pathways). Figure S15. Gene orthologs associated with carbon fixation via the Calvin cycle in phototrophic MAGs. Figure S16. Gene orthologs associated with photosynthesis in phototrophic MAGs. Figure S17. Gene orthologs associated with photosynthetic antenna proteins in phototrophic MAGs.

## Data Availability

All metagenomic and metatranscriptomic sequencing raw data, alongside MAGs assemblies, are available under the NCBI Bioproject PRJNA1273015). Protein sequences raw data are available under the PRIDE project PXD067889. Codes and metadata used in this study are available on GitHub: RIVER-EPFL/Drought-multiomics-biofilms.
