# Supplementary material for: Experimental insights in taxon-specific functional responses to droughts in glacier-fed stream biofilms
Source: Microbiome. 2026 Feb 11;14:65. doi: 10.1186/s40168-026-02336-6 (PMC12896324; doi:10.1186/s40168-026-02336-6)
Supplement: Supplementary file 2 — Supplementary Material 1: Table S1. Sequencing and processing statistics of the metagenomics samples. Table S2. Summary statistics of the metagenome (co)assemblies. Table S3. Sequencing and processing statistics of the metatranscriptomic samples. [file 40168_2026_2336_MOESM1_ESM.pdf]

Supplementary Table 1. Sequencing and processing statistics of the metagenomic samples.

Sample ID	Sequencing platform	Raw reads	Passed QC reads	Total length (bp)	NCBI Accession #
S1_metagenome	Element Aviti	17,975,886	17,872,628	2,538,312,310	SRR33970704
	ONT minION		129,164	252,770,999	SRR33985142
S2_metagenome	Element Aviti	22,422,592	22,364,439	3,157,104,717	SRR33970703
	ONT minION		384,367	599,171,501	SRR33985141
S3_metagenome	Element Aviti	19,871,805	19,788,741	2,803,403,493	SRR33970692
	ONT minION		890,794	1,566,941,961	SRR33985130
S4_metagenome	Element Aviti	20,023,429	19,929,781	2,817,515,123	SRR33970689
	ONT minION		837,262	2,088,054,664	SRR33985127
S5_metagenome	Element Aviti	15,897,467	15,807,219	2,274,222,522	SRR33970688
	ONT minION		495,504	1,003,292,940	SRR33985126
S6_metagenome	Element Aviti	21,651,088	21,535,381	3,051,412,712	SRR33970687
	ONT minION		866,793	1,746,519,947	SRR33985125
S7_metagenome	Element Aviti	24,243,137	24,116,262	3,401,833,314	SRR33970686
	ONT minION		398,697	837,192,731	SRR33985124
S8_metagenome	Element Aviti	19,664,635	19,530,878	2,791,386,607	SRR33970685
	ONT minION		33,020	45,858,303	SRR33985123
S9_metagenome	Element Aviti	22,881,685	22,759,355	3,160,180,411	SRR33970684
	ONT minION		770,448	1,579,702,008	SRR33985122
S10_metagenome	Element Aviti	28,974,799	28,780,035	3,758,004,607	SRR33970683
	ONT minION		203,670	271,335,874	SRR33985121
S11_metagenome	Element Aviti	17,054,322	16,968,763	2,337,166,430	SRR33970702
	ONT minION		246,308	478,367,498	SRR33985140
S12_metagenome	Element Aviti	19,952,050	19,839,291	2,782,704,132	SRR33970701
	ONT minION		436,101	991,821,806	SRR33985139
S13_metagenome	Element Aviti	18,989,949	18,841,988	2,658,329,398	SRR33970700
	ONT minION		522,361	890,795,698	SRR33985138
S14_metagenome	Element Aviti	20,067,677	19,953,755	2,784,544,163	SRR33970699
	ONT minION		575,322	1,042,730,089	SRR33985137
S15_metagenome	Element Aviti	17,250,042	17,139,494	2,427,442,022	SRR33970698
	ONT minION		315,795	459,883,830	SRR33985136
S16_metagenome	Element Aviti	23,478,780	23,363,081	3,268,764,035	SRR33970697
	ONT minION		195,395	316,739,893	SRR33985135
S17_metagenome	Element Aviti	20,460,249	20,370,742	2,839,249,607	SRR33970696
	ONT minION		650,679	1,040,296,297	SRR33985134
S18_metagenome	Element Aviti	18,071,477	18,006,121	2,490,501,344	SRR33970695
	ONT minION		1,083,545	1,705,852,807	SRR33985133
S19_metagenome	Element Aviti	17,383,201	17,305,707	2,404,903,831	SRR33970694
	ONT minION		819,916	1,324,661,297	SRR33985132
S20_metagenome	Element Aviti	21,120,995	21,017,911	2,907,972,074	SRR33970693
	ONT minION		874,956	1,457,187,365	SRR33985131
S21_metagenome	Element Aviti	20,705,513	20,627,062	2,833,099,359	SRR33970691
	ONT minION		25,420	18,834,578	SRR33985129
S22_metagenome	Element Aviti	20,552,412	20,470,741	2,845,904,317	SRR33970690
	ONT minION		196,092	240,493,793	SRR33985128
NEG_metagenome	Element Aviti	8,064	6,933	952,619	SRR34400844

Supplementary Table 2. Summary statistics of the metagenome (co)assemblies.

Assembly ID	Number of contigs	Total length (bp)	GC %	N50
Co_all_aviti	1,085,346	2,323,469,736	49.39	2,217
Before_aviti	493,902	1,068,958,779	46.79	2,267
After_aviti	618,692	1,290,359,328	51.95	2,267
P1_aviti	212,671	457,473,019	47.59	2,275
P2_aviti	244,194	514,304,726	46.17	2,090
P3_aviti	203,092	408,538,361	51.44	2,042
P4_aviti	243,488	521,182,794	53.28	2,076
P5_aviti	227,039	457,174,042	50.69	1,957
Co_all_minION	116,784	957,240,678	51.33	10,891

Supplementary Table 3. Sequencing and processing statistics of the metatranscriptomic samples.

Sample ID	Raw reads	Passed QC reads	Sorted reads	Total length (bp)	NCBI Accession #
S1_ metatranscriptome	20,233,008	20,154,069	6,773,832	990,336,796	SRR34006962
S2_ metatranscriptome	64,620,637	64,453,363	17,982,114	2,627,708,824	SRR34006961
S3_ metatranscriptome	52,525,451	52,073,690	8,782,096	1,262,314,141	SRR34006952
S4_ metatranscriptome	53,050,352	52,045,919	12,262,238	1,758,775,896	SRR34006951
S5_ metatranscriptome	18,292,254	18,230,911	4,085,434	600,598,287	SRR34006950
S6_ metatranscriptome	55,727,641	55,578,869	9,483,251	1,371,565,547	SRR34006949
S7_ metatranscriptome	42,423,793	42,033,225	13,118,815	1,902,336,119	SRR34006948
S8_ metatranscriptome	46,495,225	46,364,083	14,705,683	2,165,308,818	SRR34006947
S9_ metatranscriptome	20,812,673	20,693,264	3,920,999	564,414,613	SRR34006946
S10_ metatranscriptome	42,487,093	42,314,597	9,347,866	1,258,805,225	SRR34006945
S11_ metatranscriptome	31,127,613	30,155,685	10,557,139	1,254,181,055	SRR34006960
S12_ metatranscriptome	42,559,184	42,086,345	5,488,240	745,707,188	SRR34006959
S13_ metatranscriptome	16,364,398	16,274,759	3,723,548	519,220,875	SRR34006958
S14_ metatranscriptome	17,808,665	17,707,449	7,738,535	1,083,007,447	SRR34006957
S15_ metatranscriptome	22,401,834	22,013,778	4,793,773	673,334,360	SRR34006956
S16_ metatranscriptome	18,725,893	18,322,687	5,726,269	749,951,171	SRR34006955
S17_ metatranscriptome	1,218,748	1,177,389	828,505	103,974,090	SRR34006954
S18_ metatranscriptome	28,197,374	28,123,379	4,526,202	662,206,706	SRR34006953
NEG_metatranscriptome	1,354,971	1,232,745	345,270	45,438,011	SRR34400843
