# Supplementary material for: Experimental insights in taxon-specific functional responses to droughts in glacier-fed stream biofilms
Source: Microbiome. 2026 Feb 11;14:65. doi: 10.1186/s40168-026-02336-6 (PMC12896324; doi:10.1186/s40168-026-02336-6)
Supplement: Supplementary file 4 — Supplementary Material 3: Figure S1. Quality and characteristics summary of bacterial MAGs recovered from a glacier-fed stream biofilm growth experiment under repetitive droughts. Figure S2. Eukaryotic MAGs recovered from a glacier-fed stream biofilm growth experiment under repetitive droughts. Figure S3. Microbial composition of whole glacier-fed stream biofilm metagenomes. Figure S4. Successional patterns in glacier-fed stream biofilm microbiomes under repetitive droughts. Figure S5. Taxonomy and successional patterns of DNA and RNA viral MAGs in glacier-fed stream biofilm microbiomes under repetitive droughts. Figure S6. Drought-induced changes in MAG activity (metatranscriptome) of glacier-fed stream biofilms. Figure S7. Drought-induced changes in activity (whole metatranscriptome) in glacier-fed stream biofilms. Figure S8. Drought-induced changes in glacier-fed stream biofilm MAGs’ activity. Figure S9. Principal component analysis (PCA) plot of variance-stabilizing transformed counts, showing clustering of replicate samples. Figure S10. Differential abundance analyses showing the taxon-scaled differentially expressed genes between pre- and post-drought samples. Figure S11. Drought-induced relative changes in MAGs’ activity. Figure S12. Number of drought-induced DEGs per KEGG metabolic category for each drought period. Figure S13. Drought-induced relative changes in biofilm functionality (KEGG subcategories). Figure S14. Drought-induced relative changes in biofilm functionality (KEGG pathways). Figure S15. Gene orthologs associated with carbon fixation via the Calvin cycle in phototrophic MAGs. Figure S16. Gene orthologs associated with photosynthesis in phototrophic MAGs. Figure S17. Gene orthologs associated with photosynthetic antenna proteins in phototrophic MAGs. [file 40168_2026_2336_MOESM3_ESM.pdf]

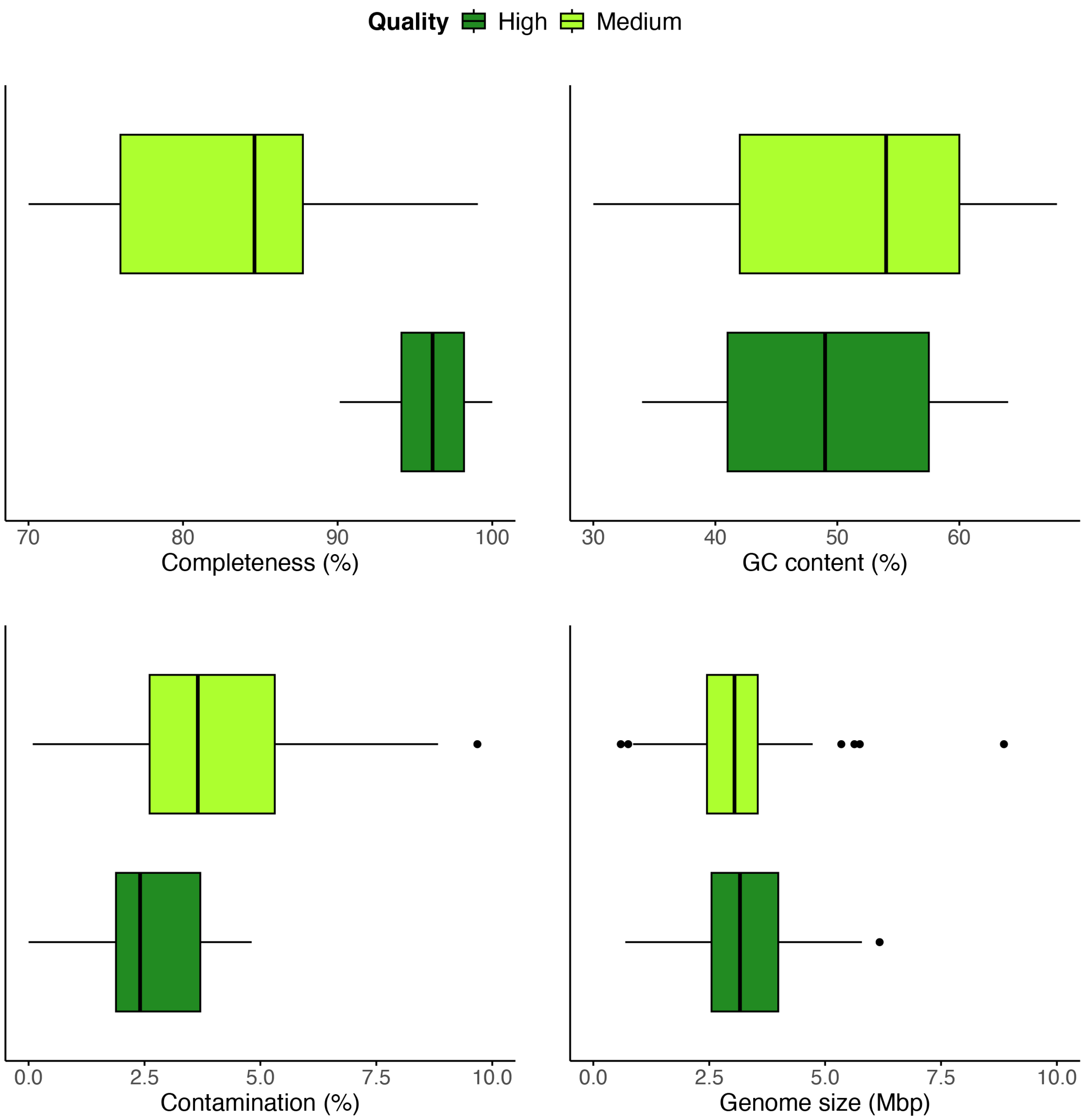

Figure S1. Quality and characteristics summary of bacterial MAGs recovered from a glacier-fed stream biofilm growth experiment under repetitive droughts.
Boxplots display of the completeness, contamination, GC content, and genome size of bacterial MAGs. MAGs are categorized based on their quality: high (completeness > 90%, contamination < 5%) or medium (completeness > 70%, contamination < 10%).

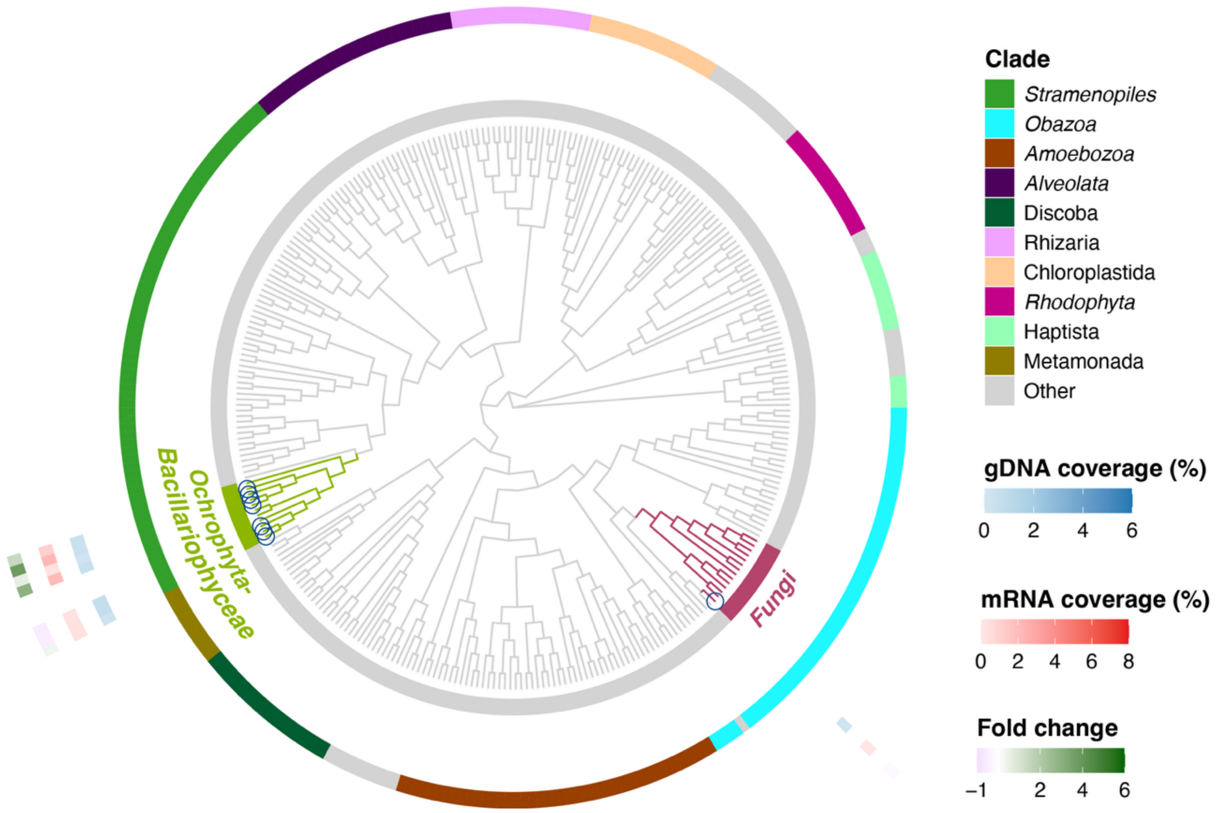

Figure S2. Eukaryotic MAGs recovered from a glacier-fed stream biofilm growth experiment under repetitive droughts.

Maximum likelihood phylogenetic tree of eukaryotic MAGs. The branches and inner ring colors correspond to the taxonomy of eukaryotic MAGs at the phylum level. The second ring colors indicate the taxonomy at the clade level. Branches marked with a blue circle indicated the MAGs recovered in this study. Other branches were included to construct the tree and assign taxonomy to the MAGs. The blue gradient color ring shows the relative metagenomic coverage (gDNA) of each MAG. The red gradient color ring shows the relative metatranscriptomic coverage (mRNA) of each MAG. The ring with a gradient color from pink to green represents the fold change between gDNA and mRNA. A green color indicates relatively more transcripts than genomic material, meaning a overrepresentation of the MAG in the metatranscriptome.

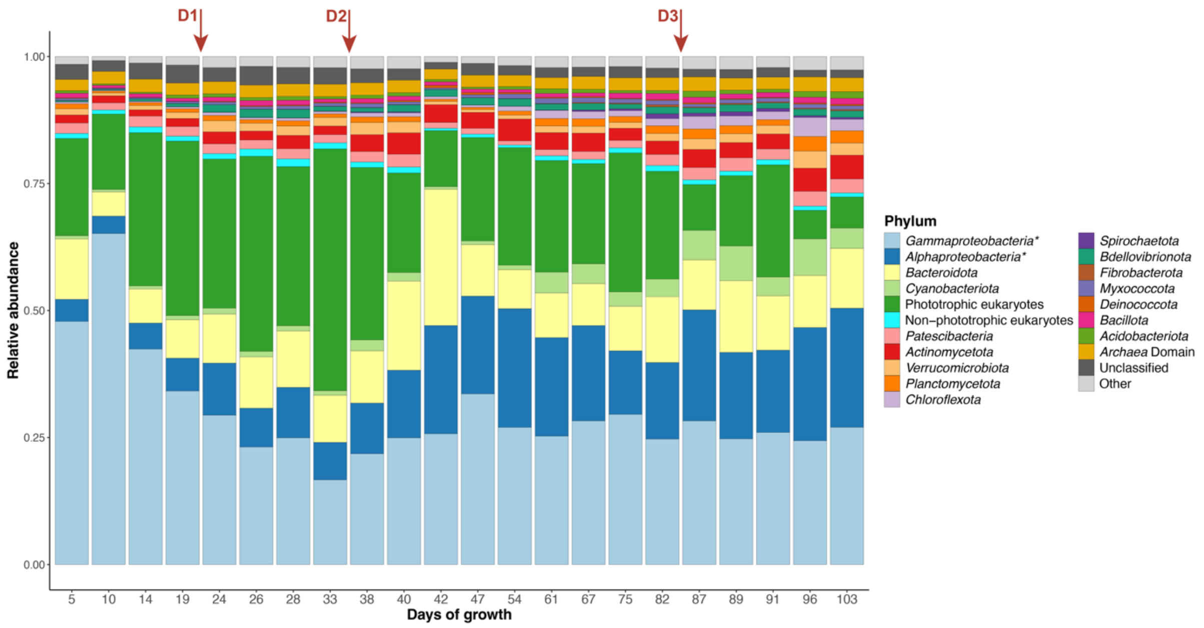

Figure S3. Microbial composition of whole glacier-fed stream biofilm metagenomes. Microbial community composition of glacier-fed stream metagenomic reads along the successional period. Reads' taxonomy was assigned using *SingleM* (Woodcroft et al. 2025), where eukaryotic reads were divided into phototrophic and non-phototrophic groups. *Cryptophyceae*, *Glaucocystophyceae*, Haptista, *Rhodophyta*, Sar, and *Viridiplantae* phyla were considered as phototrophic eukaryotes. D1, D2, and D3 indicate the timing of the three droughts.* *Gammaproteobacteria* and *Alphaproteobacteria* are classes of the *Pseudomonadota* phylum.

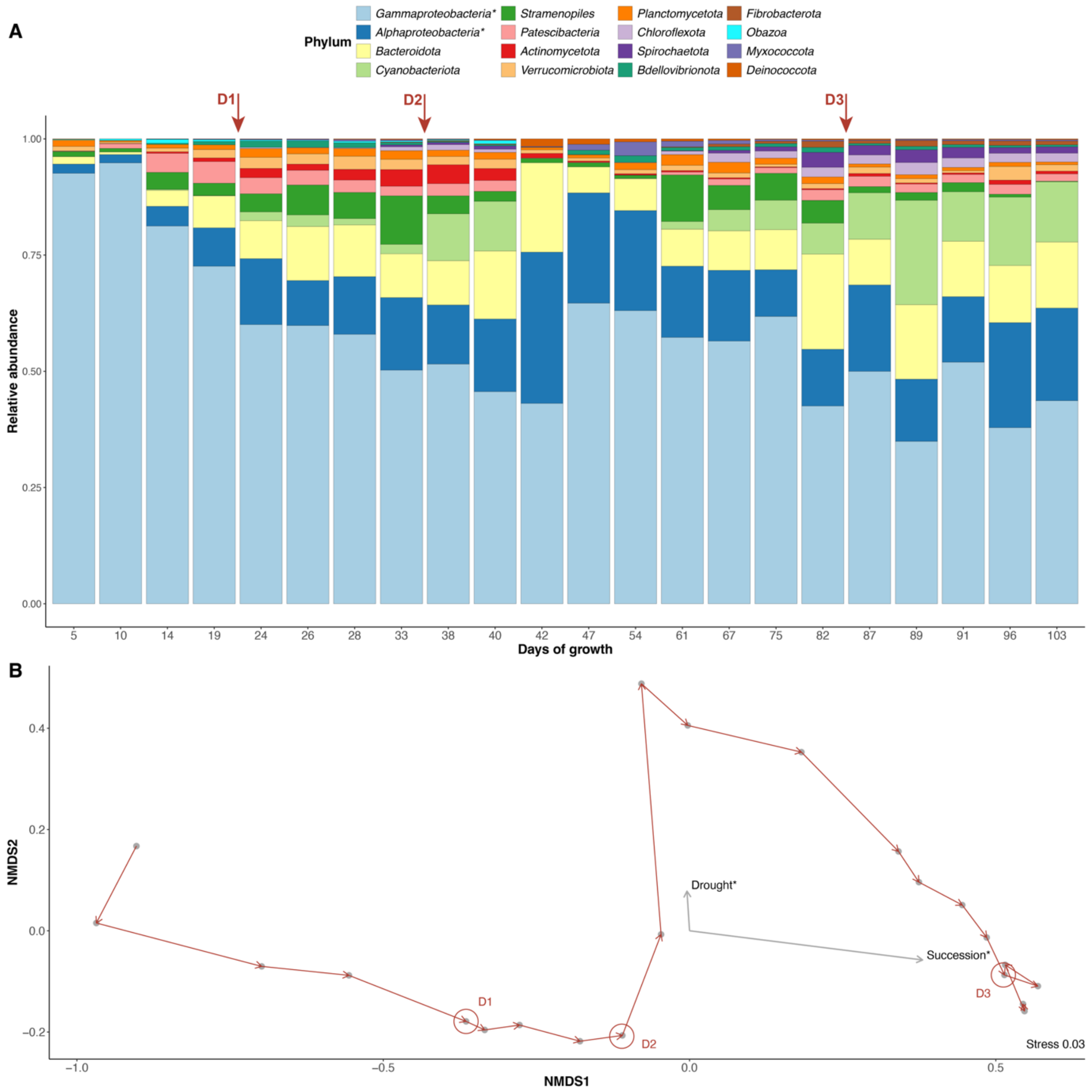

Figure S4. Successional patterns in glacier-fed stream biofilm microbiomes under repetitive droughts.

(A) Community composition of the bacterial (phylum) and eukaryotic (clade) MAGs, for each metagenomic sample. (B) Changes in MAG community composition are shown by a nonmetric multidimensional scaling (NMDS) ordination based on Bray-Curtis dissimilarity. Arrows show parameters significantly explaining changes in community composition ($p < 0.05$). D1, D2, and D3 indicate the timing of the three droughts. * *Gammaproteobacteria* and *Alphaproteobacteria* are classes of the *Pseudomonadota* phylum.

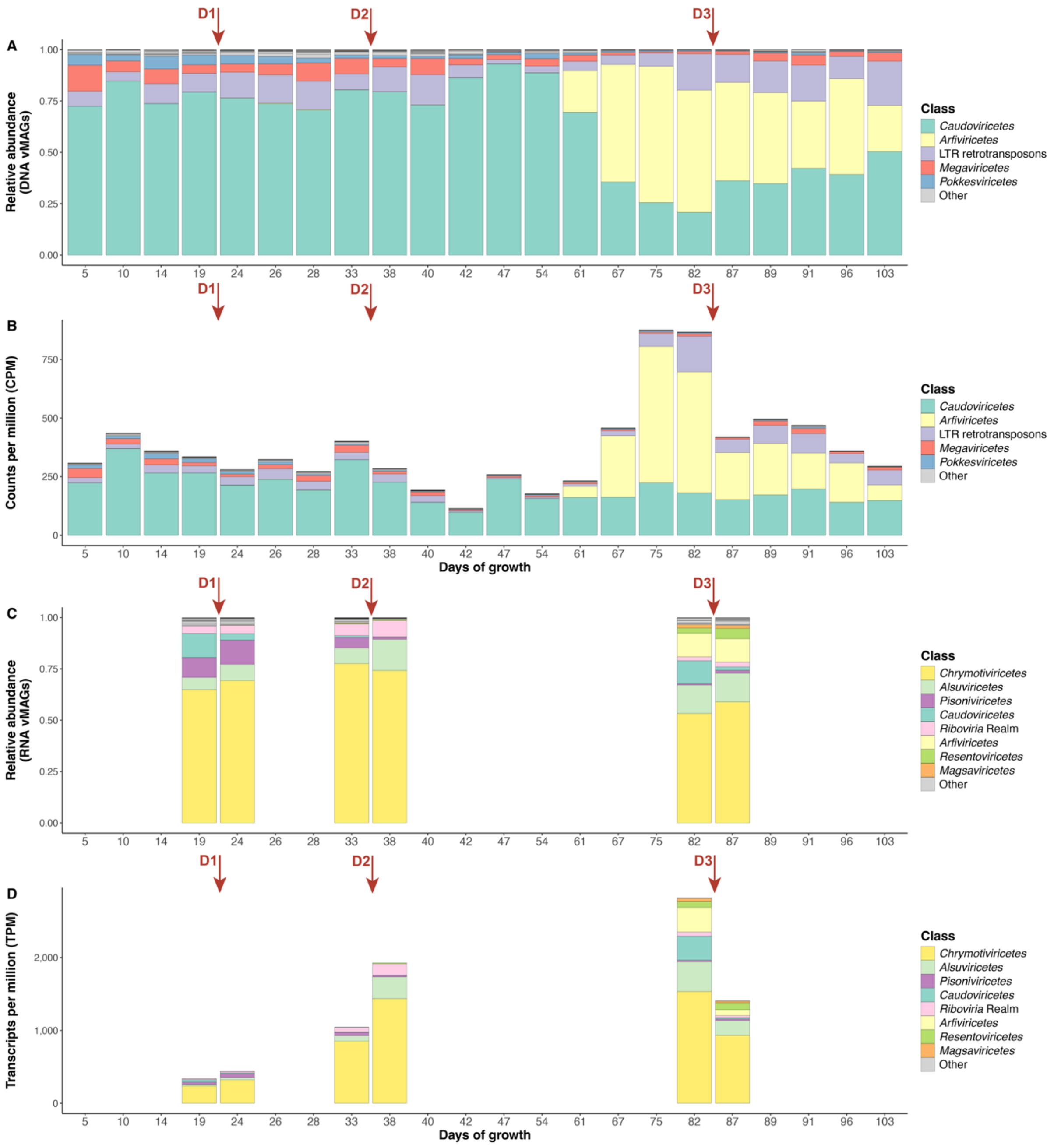

Figure S5. Taxonomy and successional patterns of DNA and RNA viral MAGs in glacier-fed stream biofilm microbiomes under repetitive droughts.

Composition of DNA-derived viral MAGs at the class level, in relative abundance (A) and counts per million (B). Composition of RNA-derived viral MAGs at the class level, in relative abundance (C) and counts per million (D), where replicate samples were merged for visualization. D1, D2, and D3 indicate the timing of the three droughts.

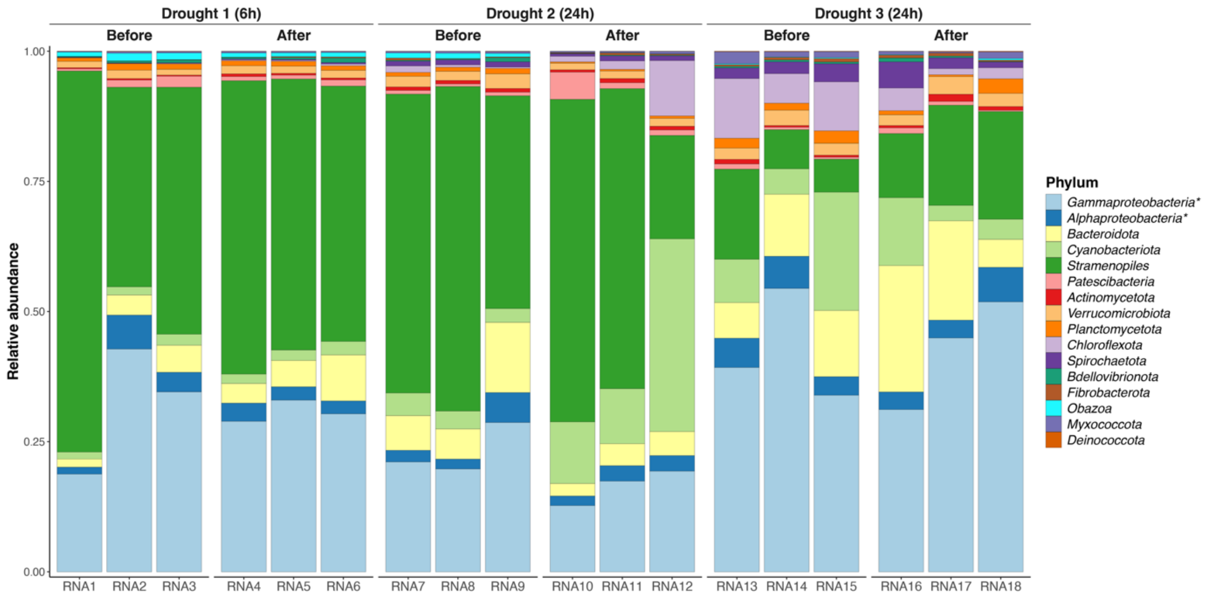

Figure S6. Drought-induced changes in MAG activity (metatranscriptome) of glacier-fed stream biofilms.

Relative abundance of transcripts (i.e., activity) assigned to MAGs within the individual metatranscriptomic samples, pre- and post-droughts. * *Gammaproteobacteria* and *Alphaproteobacteria* are classes of the *Pseudomonadota* phylum.

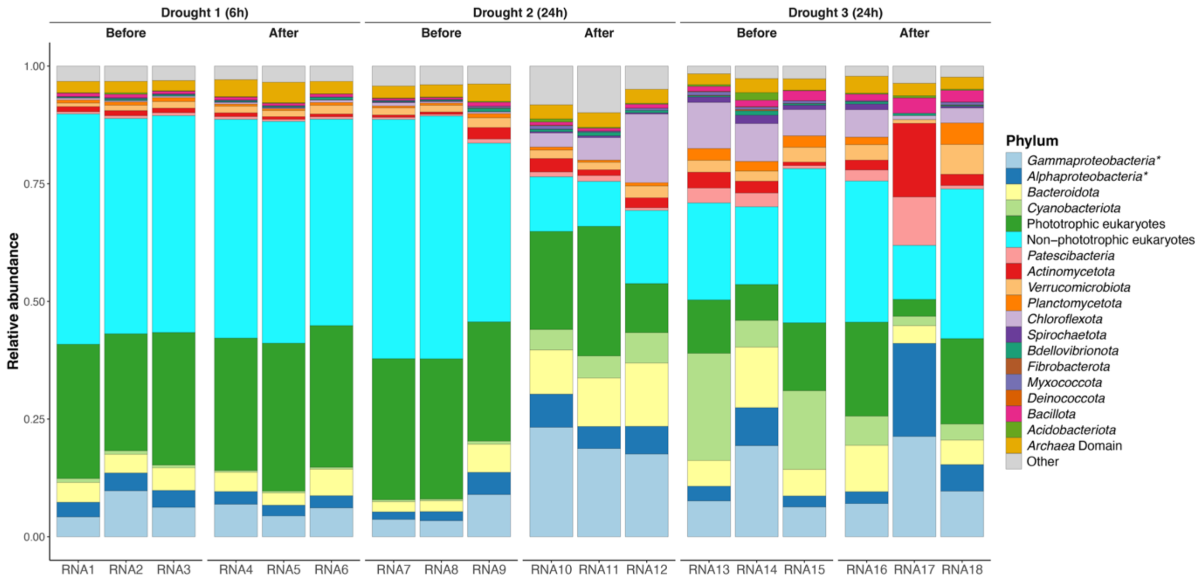

Figure S7. Drought-induced changes in activity (whole metatranscriptome) in glacier-fed stream biofilms.

Relative abundance of the whole mRNA reads (i.e., activity) within the pre- and post-drought metatranscriptomes. Metaranscriptomic replicate samples were merged for visualization. Reads' taxonomy was assigned using *SingleM* (Woodcroft et al. 2025), where eukaryotic reads were divided into phototrophic and non-phototrophic groups. *Cryptophyceae*, *Glaucocystophyceae*, Haptista, *Rhodophyta*, Sar, and *Viridiplantae* phyla were considered as phototrophic eukaryotes. * *Gammaproteobacteria* and *Alphaproteobacteria* are classes of the *Pseudomonadota* phylum.

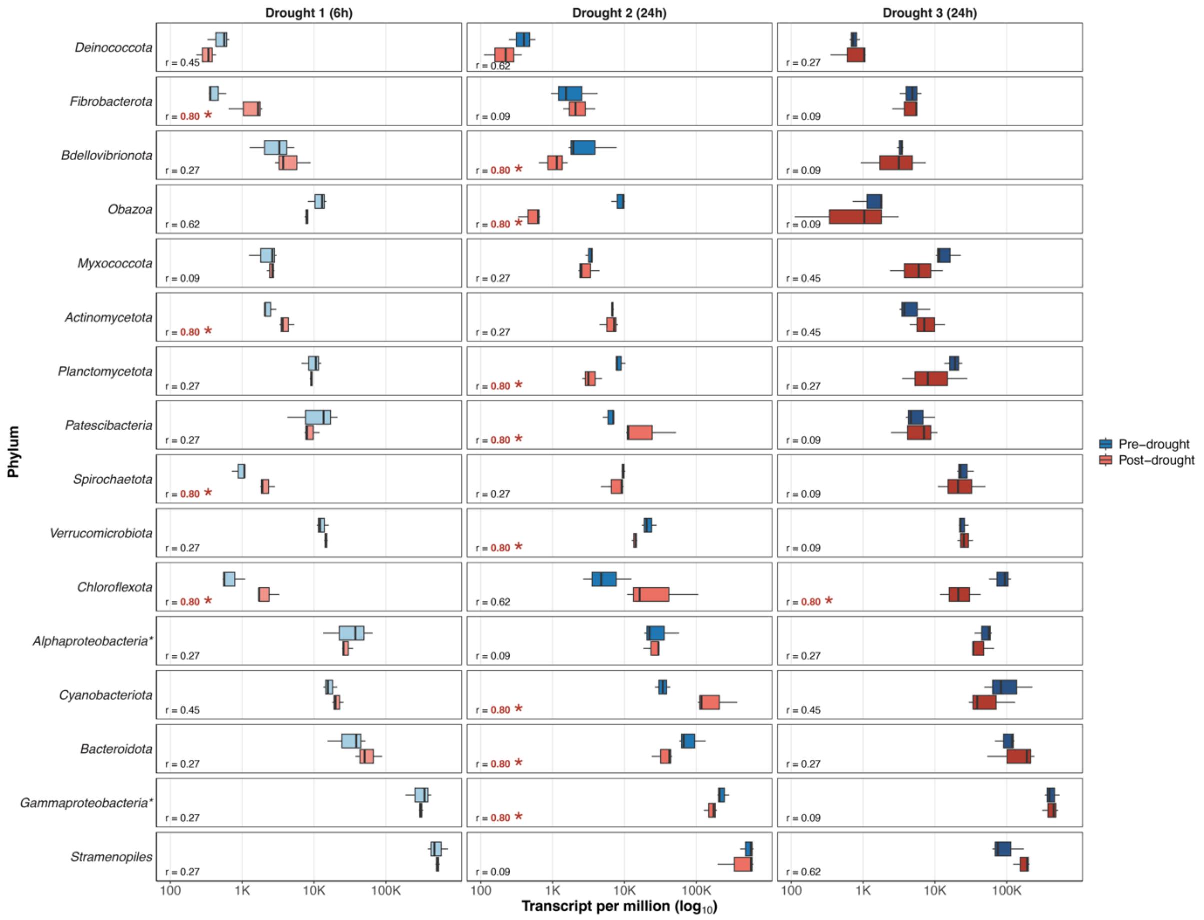

Figure S8. Drought-induced changes in glacier-fed stream biofilm MAGs' activity. Differences in relative activity between pre- and post-drought conditions for each drought period, for the most abundant MAG taxonomic groups. "r" is the standardized effect size between the two conditions. Bolded red text with a star (*) indicates a significant difference ($p < 0.05$) in relative activity between pre- and post-drought conditions for each drought period. * *Gammaproteobacteria* and *Alphaproteobacteria* are classes of the *Pseudomonadota* phylum.

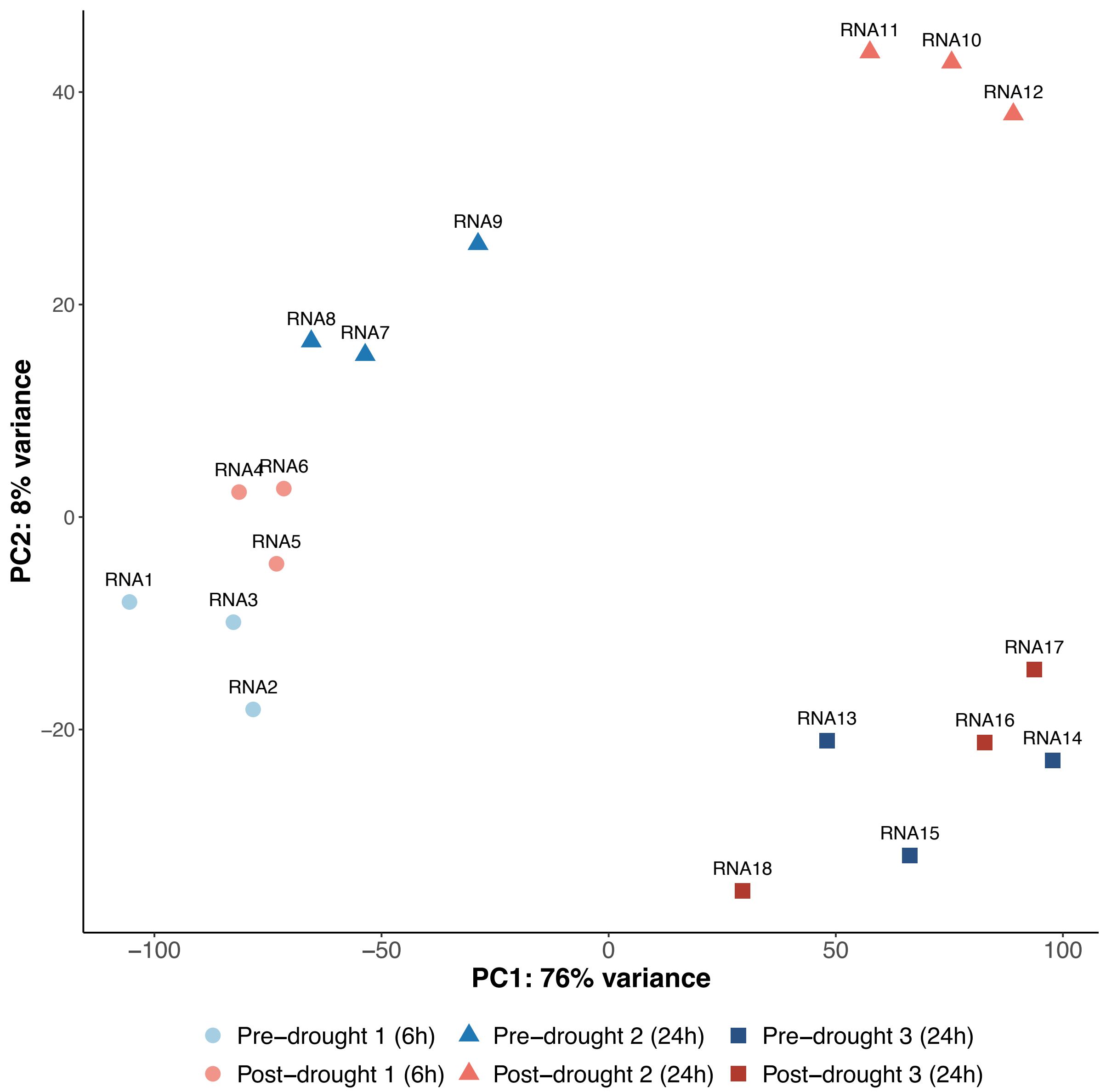

Figure S9. Principal component analysis (PCA) plot of variance-stabilizing transformed counts, showing clustering of replicate samples.
The PCA reveals clustering of metatranscriptomic samples by replicates, with differentiation over time and between pre- and post-drought conditions.

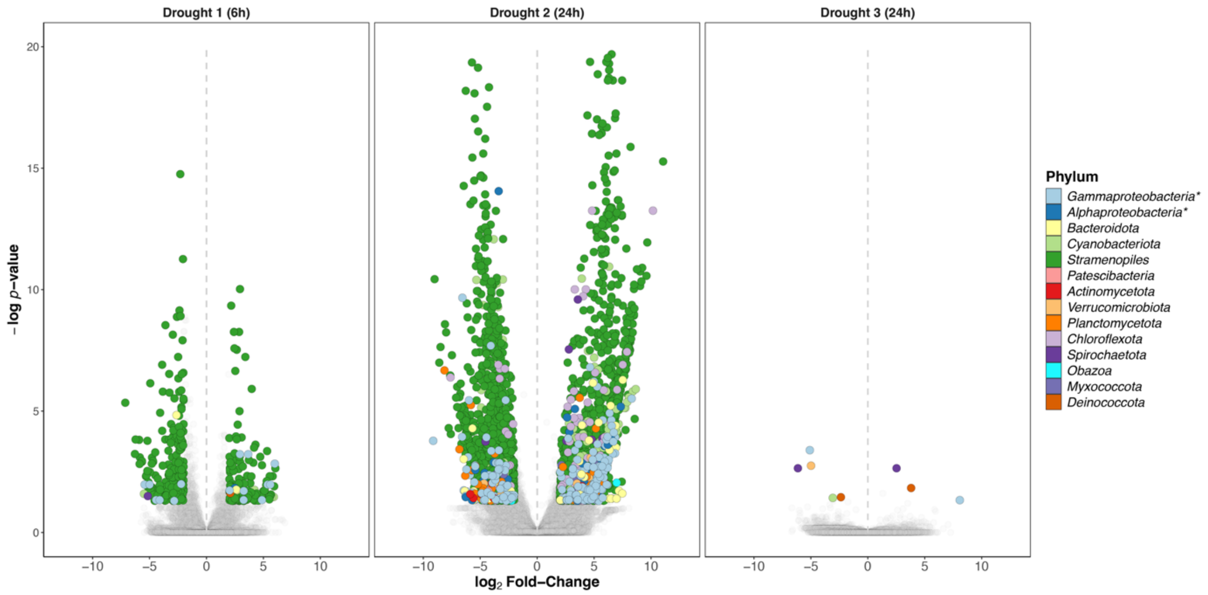

Figure S10. Differential abundance analyses showing the taxon-scaled differentially expressed genes between pre- and post-drought samples.

Taxon-scaled drought-induced significant differentially expressed genes (DEGs), where positive log_2 fold-change indicates up-regulated genes after a drought event (right of the dashed line), and negative log_2 fold-change indicates down-regulated genes after a drought event (left of the dashed line). DEGs are color-coded by taxonomy of their MAG of origin (phylum level). * *Gammaproteobacteria* and *Alphaproteobacteria* are classes of the *Pseudomonadota* phylum.

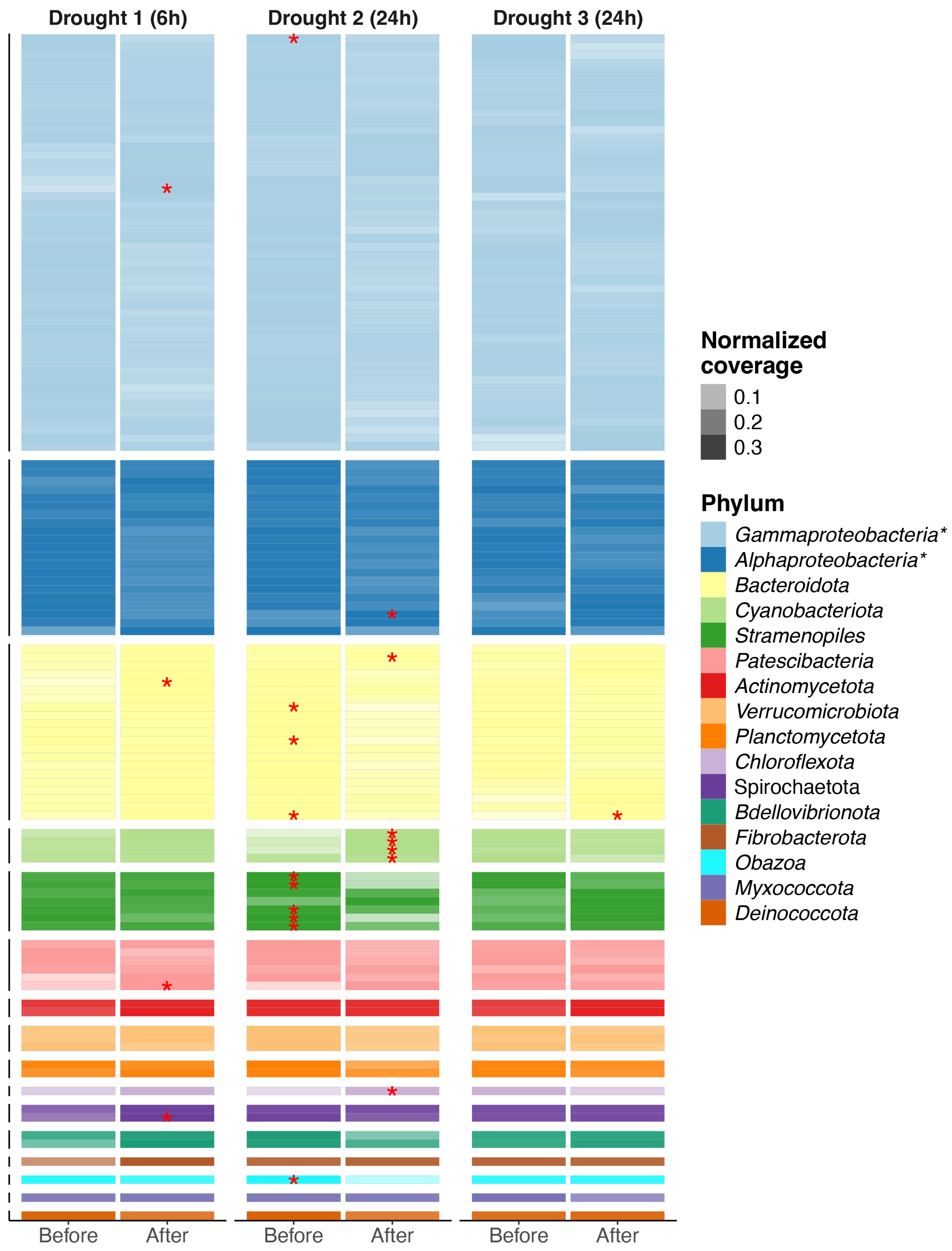

Figure S11. Drought-induced relative changes in MAGs' activity.
MAG's relative change in activity between pre- and post-drought conditions for each drought period. Each line represents a different MAG, color-coded by phylum. Red stars indicate significantly differentially abundant MAGs identified in Figure 3. * *Gammaproteobacteria* and *Alphaproteobacteria* are classes of the *Pseudomonadota* phylum.

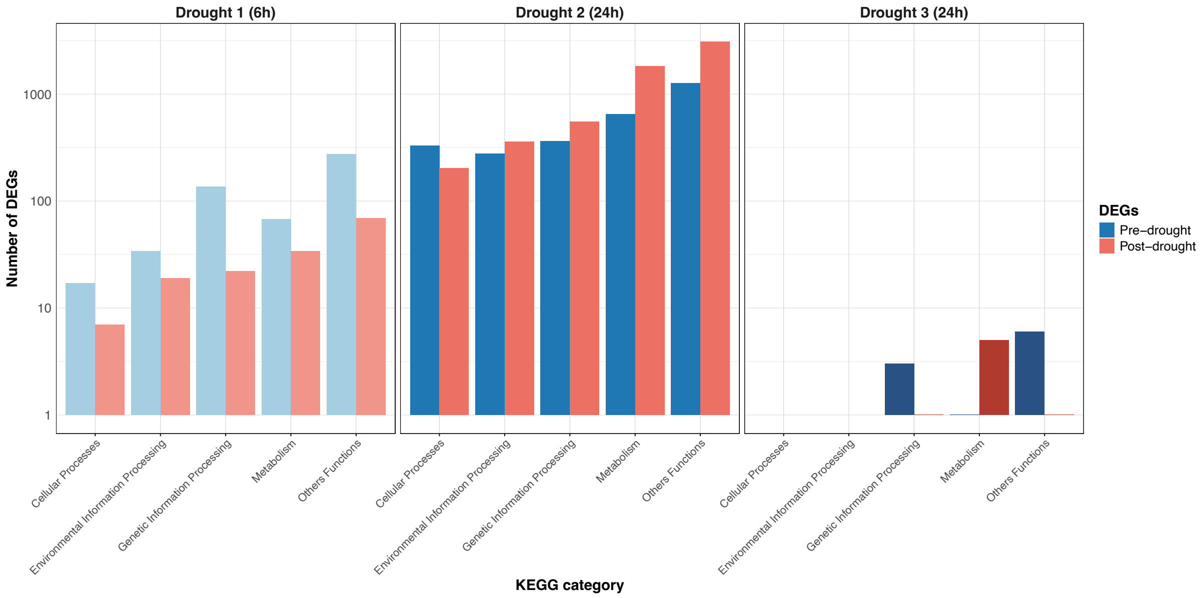

Figure S12. Number of drought-induced DEGs per KEGG metabolic category for each drought period.

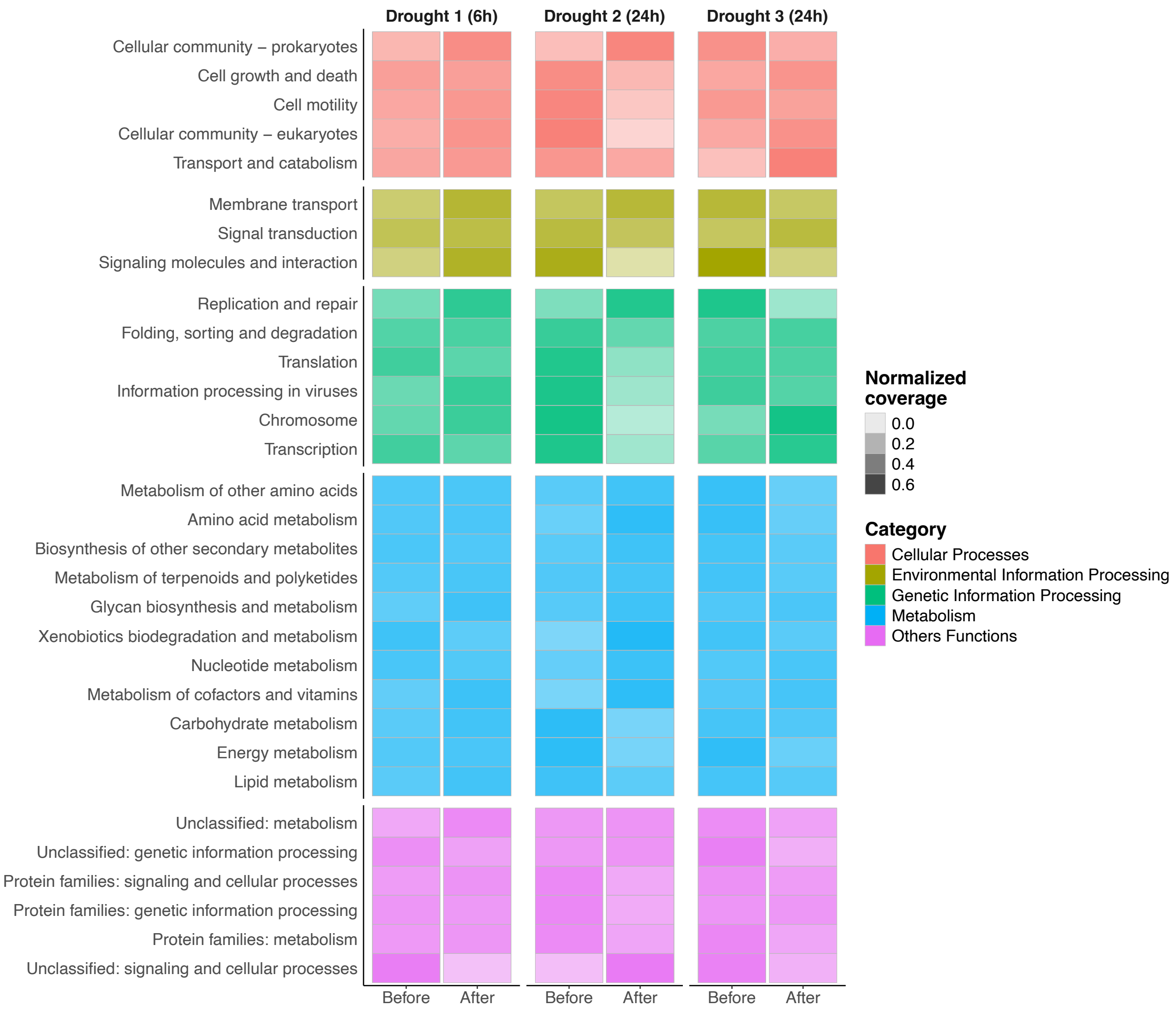

Figure S13. Drought-induced relative changes in biofilm functionality (KEGG subcategories).

Glacier-fed stream biofilm's relative change in functionality (coverage of DEGs) between pre- and post-drought conditions for each drought period. Each line represents a KEGG functional subcategory, color-coded by category.

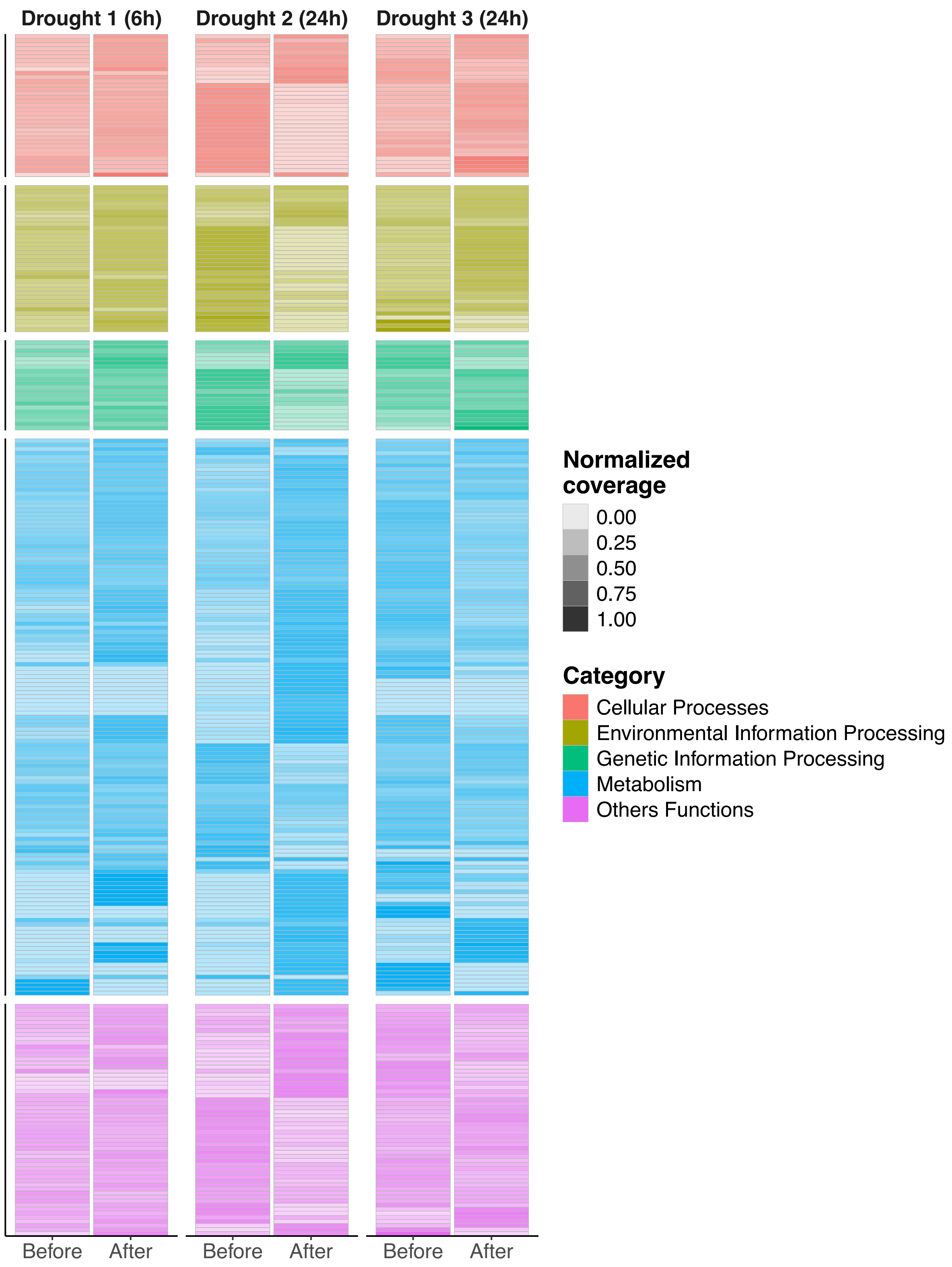

Figure S14. Drought-induced relative changes in biofilm functionality (KEGG pathways). Glacier-fed stream biofilm's relative change in functionality (coverage of DEGs) between pre- and post-drought conditions for each drought period. Each line represents a KEGG functional pathway, color-coded by category.

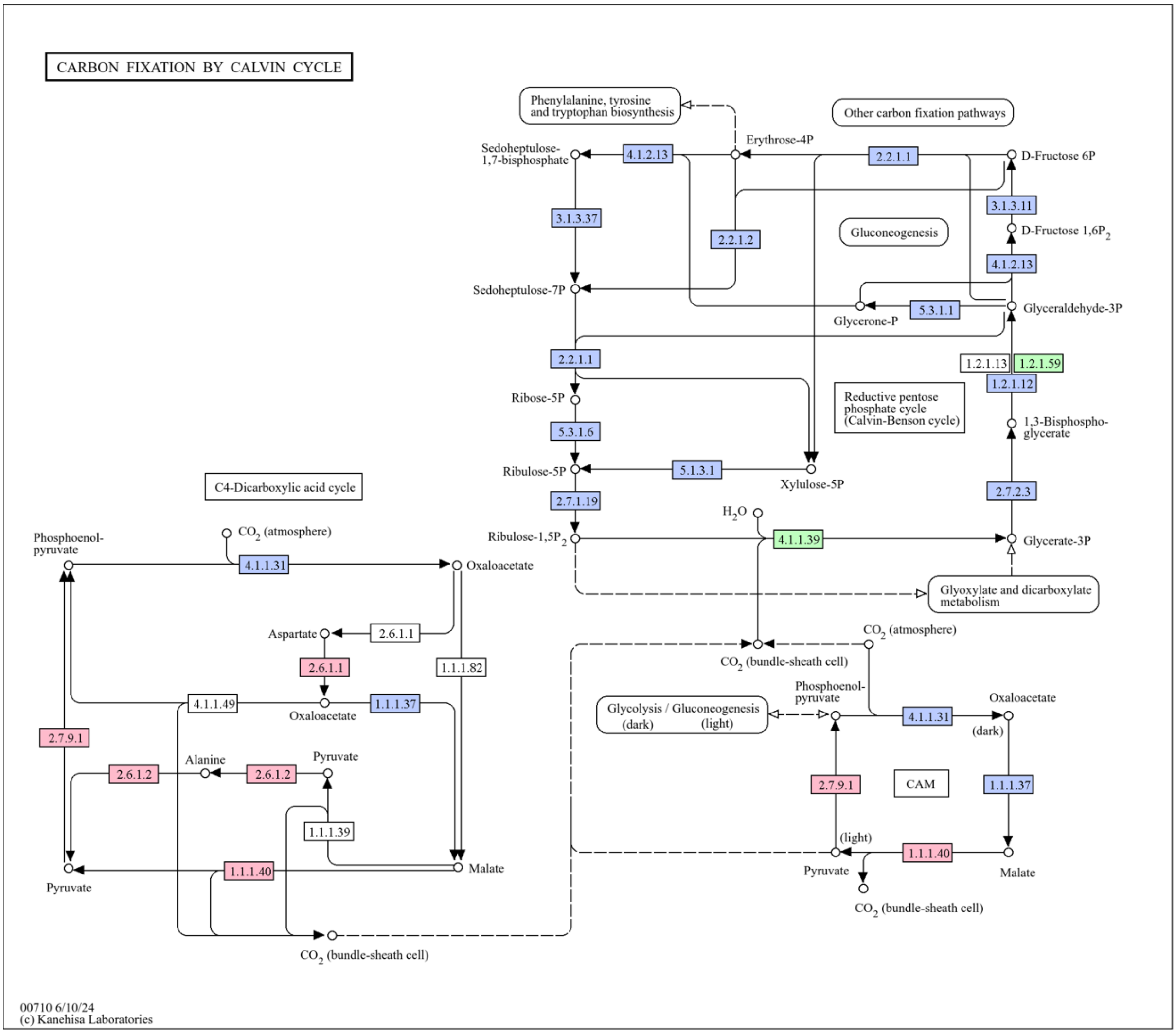

Figure S15. Gene orthologs associated with carbon fixation via the Calvin cycle in phototrophic MAGs.

KEGG orthologs (KOs) detected in cyanobacterial MAGs (green), diatom MAGs (red), or shared between both groups (purple). Image downloaded from KEGG Mapper – Reconstruct (Kanehisa, 2017; https://www.genome.jp/kegg/mapper/reconstruct.html).

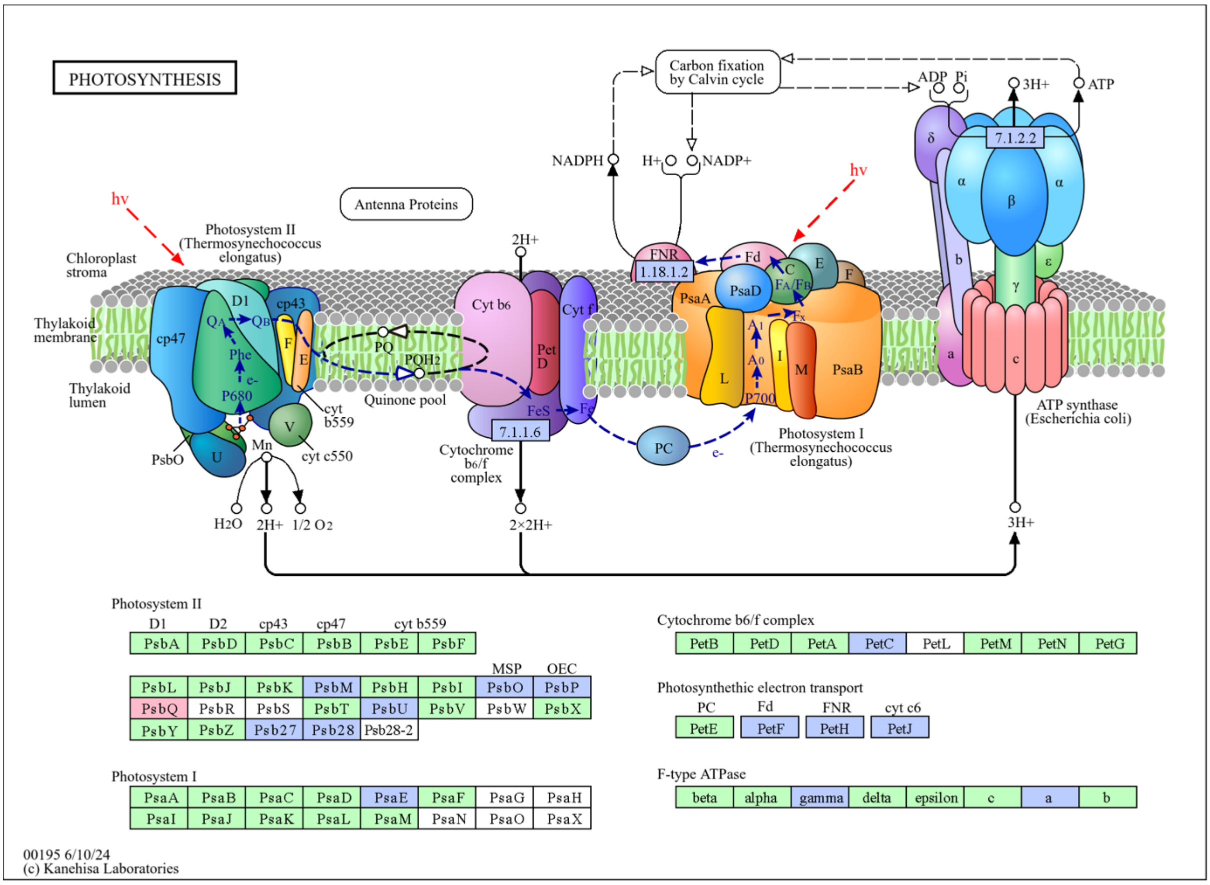

Figure S16. Gene orthologs associated with photosynthesis in phototrophic MAGs. KEGG orthologs (KOs) detected in cyanobacterial MAGs (green), diatom MAGs (red), or shared between both groups (purple). Image downloaded from KEGG Mapper – Reconstruct (Kanehisa, 2017; https://www.genome.jp/kegg/mapper/reconstruct.html).

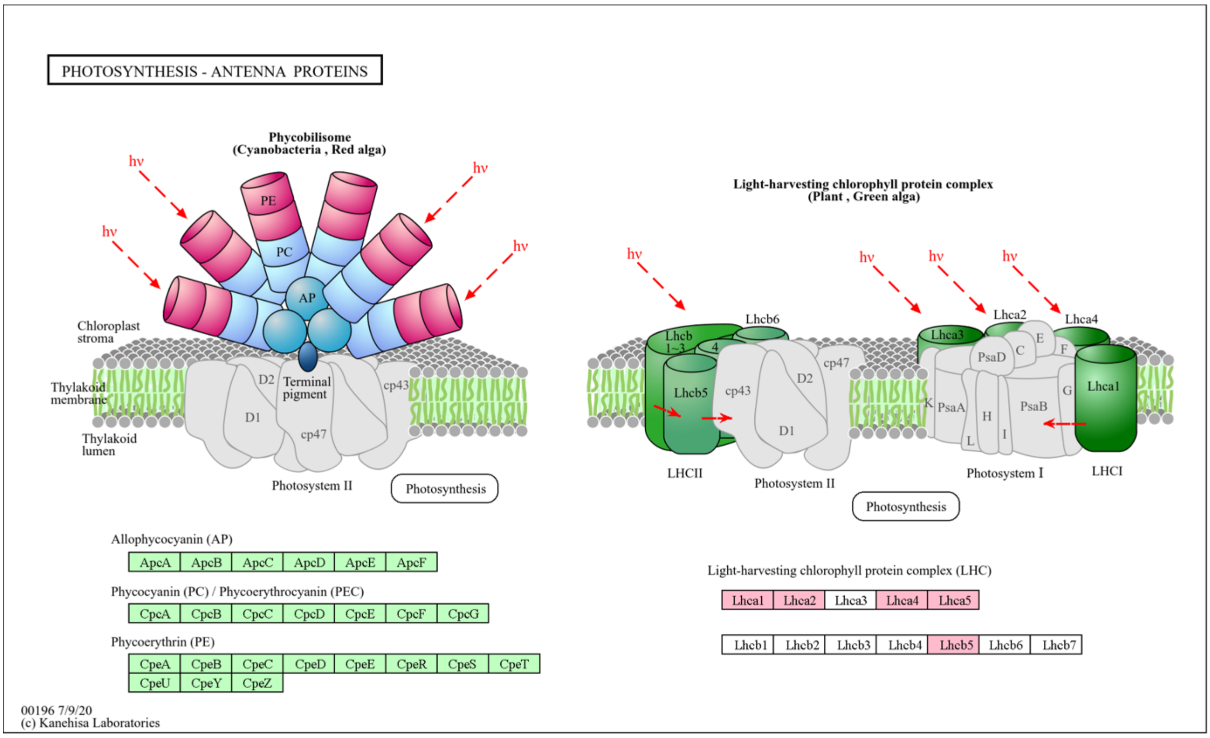

Figure S17. Gene orthologs associated with photosynthetic antenna proteins in phototrophic MAGs.

KEGG orthologs (KOs) detected in cyanobacterial MAGs (green), diatom MAGs (red), or shared between both groups (purple). Image downloaded from KEGG Mapper – Reconstruct (Kanehisa, 2017; https://www.genome.jp/kegg/mapper/reconstruct.html).

References

Woodcroft, Ben J., Samuel T. N. Aroney, Rossen Zhao, et al. 2025. 'Comprehensive Taxonomic Identification of Microbial Species in Metagenomic Data Using SingleM and Sandpiper'. Nature Biotechnology, July 16, 1–6. https://doi.org/10.1038/s41587-025-02738-1.

Kanehisa M. Enzyme Annotation and Metabolic Reconstruction Using KEGG. In: Kihara D, editor. Protein Function Prediction: Methods and Protocols. New York, NY: Springer; 2017. p. 135–45. https://doi.org/10.1007/978-1-4939-7015-5_11.
